# Phase 1 study of ceralasertib, an ATR kinase inhibitor, in combination with durvalumab in patients with recurrent or metastatic NSCLC or HNSCC

**DOI:** 10.1038/s41416-026-03408-y

**Published:** 2026-03-31

**Authors:** Juanita S. Lopez, Kevin J. Harrington, Seock-Ah Im, Keun-Wook Lee, Sophie Postel-Vinay, Jacob S. Thomas, Natalia Lukashchuk, Sophie E. Willis, Itziar Irurzun-Arana, Benjamin Webb, Jyoti Nehra, Alan Lau, Arsène-Bienvenu Loembé, Emma Dean, Matthew G. Krebs

**Affiliations:** 1 Drug Development Unit, The Royal Marsden Hospital and The Institute of Cancer Research, Sutton, UK; 2 The Institute of Cancer Research and The Royal Marsden NHS Foundation Trust National Institute of Health Research Biomedical Research Centre, London, UK; 3 Seoul National University Hospital, Cancer Research Institute, Seoul National University College of Medicine, Seoul, Republic of Korea; 4 Seoul National University College of Medicine, Seoul National University Bundang Hospital, Seongnam, Republic of Korea; 5 Institut Gustave Roussy, Villejuif, France; 6 University of Southern California Norris Comprehensive Cancer Center, Los Angeles, CA USA; 7 Translational Medicine Early Oncology, Early Oncology Development, Oncology R&D, AstraZeneca, Cambridge, UK; 8 Cancer Biomarker Development, Early Oncology Development, Oncology R&D, AstraZeneca, Cambridge, UK; 9 Clinical Pharmacology Quantitative Pharmacology, Early Oncology Development, Oncology R&D, AstraZeneca, Cambridge, UK; 10 Early Oncology Statistics, Biometrics Oncology, Oncology R&D, AstraZeneca, Cambridge, UK; 11 Late Oncology Development, GI Cancer and Strategy, Oncology R&D, AstraZeneca, Waltham, MA USA; 12 UK – SM Bioscience Team, Oncology Targeted Discovery, Oncology R&D, AstraZeneca, Cambridge, UK; 13 Early Oncology Clinical, Early Oncology Development, Oncology R&D, AstraZeneca, Cambridge, UK; 14 Early ICA Projects, Early Oncology Development, Oncology R&D, AstraZeneca, Cambridge, UK; 15 Division of Cancer Sciences, Faculty of Biology, Medicine and Health, The University of Manchester and The Christie NHS Foundation Trust, Manchester, UK; 16 Present Address: GSK, Madrid, Spain; 17 Present Address: Experimental Medicine Oncology department, Boehringer-Ingelheim, Ingelheim, Germany

**Keywords:** Non-small-cell lung cancer, Cancer immunotherapy, Head and neck cancer, Head and neck cancer, Non-small-cell lung cancer

## Abstract

**Background:**

This multicentre, modular, Phase 1 study evaluated escalating doses of ATR (ataxia telangiectasia and Rad3-related kinase) inhibitor ceralasertib plus PD-L1 inhibitor durvalumab in patients with previously treated advanced/metastatic non-small-cell lung cancer (NSCLC) or head and neck squamous cell carcinoma (HNSCC).

**Methods:**

Patients received ceralasertib 80/160/240 mg twice-daily (BID) or 320 mg once-daily (QD) for 7 (Days 22–28) or 14 (Days 15–28) days, plus durvalumab 1500 mg (Day 1), per 28-day cycle. The primary objective was to investigate the safety/tolerability of the combination.

**Results:**

Sixty patients were treated. Two patients had dose-limiting toxicities of: Grade 3 thrombocytopenia with Grade 3 anaemia (ceralasertib 320 mg QD for 14 days); and Grade 4 thrombocytopenia with Grade 3 neutropenia accompanied by systemic chest infection (ceralasertib 240 mg BID for 14 days). Overall, 59 (98.3%) patients had treatment-emergent adverse events; 31 (51.7%) had grade ≥3 events. The recommended Phase 2 dose was durvalumab 1500 mg (Day 1) plus ceralasertib 240 mg BID (Days 15–28). Five (8.3%) patients had objective responses; 31 (51.7%) had stable disease. Pharmacodynamic activity (pRAD50 increase) was observed in 10/14 paired biopsies.

**Conclusion:**

Ceralasertib plus durvalumab was tolerated and associated with antitumour activity in advanced/metastatic NSCLC and HNSCC.

**Trial Registration Number:**

NCT02264678

## Introduction

Cancer cells frequently have diminished DNA repair capability related to defects in DNA damage response (DDR) pathways [1]. The resultant DNA damage and genomic instability, referred to as ‘replication stress’, slows or stalls DNA replication [1–3]. Ataxia telangiectasia and Rad3-related (ATR) is a key kinase that responds to DNA replication stress and initiates the replication stress response. This stabilises DNA replication forks, prevents new replication origin firing and stops cell cycle progression, allowing time for DNA repair and maintaining genomic stability and viability [1, 2, 4, 5]. If ATR function is impaired (e.g., through small molecule kinase inhibitors), stalled DNA replication forks cannot be resolved, leading to double-strand breaks, accumulation of DNA damage and cancer cell death [4, 6, 7].

Ceralasertib is an oral, selective and potent ATR inhibitor [8]. Preclinical studies showed that ceralasertib monotherapy has antitumour activity in models with DDR pathway deficiencies, such as loss of ataxia telangiectasia mutated (*ATM*), or those that harbour putative drivers of replication stress, like AT-rich interactive domain-containing protein 1A (*ARID1A*) deficiency or Cyclin E1 amplification. Ceralasertib antitumour activity is enhanced in these models when combined with DNA-damaging treatments, such as cytotoxic chemotherapy, poly(ADP-ribose) polymerase inhibitors or ionising radiation plus anti-programmed cell death ligand 1 (PD-L1) therapy [7, 9–18]. Emerging clinical evidence suggests promising activity and tolerability of ceralasertib monotherapy in patients with biomarker-selected solid tumours, including those with enhanced ATR inhibitor sensitivity due to *ARID1A* deficiency [19, 20].

Encouraging clinical activity has been reported with ceralasertib in combination with PD-L1 inhibitor durvalumab in advanced/metastatic melanoma, advanced gastric cancer, and non-small-cell lung cancer (NSCLC) following anti-PD-1/PD-L1 [PD-(L)1] failure [21–23]. DDR defects and resultant replication stress may enhance or restore antitumour response to immunotherapy by increasing tumour immunogenicity [3, 24]; accumulation of somatic mutations caused by defective DDR can encode tumour-specific neoantigens in tumour cells, increasing their recognition by T cells [24, 25]. Additionally, DDR failure can result in increased cytosolic DNA, stimulating the innate immune response via activation of the cGAS–STING pathway and leading to a type-1 interferon-mediated antitumour response [24, 26, 27]. Evidence indicates intermittent ATR inhibition impacts proliferating and exhausted T cells and myeloid-derived suppressor cells to remodel and reset the tumour immune microenvironment (TIME) to a naïve or permissive state. Such immune remodelling may enhance or restore susceptibility to immune checkpoint inhibition, thereby enhancing the effects of PD-L1 blockade and overcoming resistance through increased activation of T-cells and tumour cell killing [21, 23, 28, 29].

A modular Phase 1, open-label, multicentre study is ongoing to assess the safety, tolerability, pharmacokinetics, and preliminary antitumour activity of ceralasertib plus cytotoxic chemotherapy, other novel DDR agents, or anticancer treatments in patients with advanced solid malignancies (NCT02264678). Module 1 showed preliminary evidence of antitumour activity in patients receiving ceralasertib plus carboplatin, although dosing was limited by haematological toxicity [30]. Module 2 showed preliminary evidence of antitumour activity in patients with advanced breast cancer receiving ceralasertib plus olaparib [31].

We report results from Module 3, in which escalating doses of ceralasertib were combined with durvalumab in patients with previously treated recurrent or metastatic NSCLC or head and neck squamous cell carcinoma (HNSCC) who were not considered appropriate for further standard treatment. While immunotherapies have resulted in sustained clinical benefit in these populations, many patients with recurrent/metastatic NSCLC or HNSCC who receive standard-of-care immunotherapy ± chemotherapy do not respond or eventually relapse, after which there remains an unmet need for further effective treatment options [32–34].

## Methods

### Patients

Module 3 included patients aged ≥18 years with histologically or cytologically confirmed advanced recurrent or metastatic NSCLC or HNSCC and radiologically confirmed progression after at least one previous line of treatment. Primary tumour locations included the lung, the head and neck, including nasopharynx, larynx, and trachea, and the oropharynx, hypopharynx, and oral cavity. For patients with HNSCC, the first-line regimen must have included a doublet 5-fluoropyrimidine and platinum-based regimen or immunotherapy (cetuximab was permitted). Adjuvant or neoadjuvant chemotherapy containing a doublet 5-fluoropyrimidine and platinum-based regimen was considered first-line treatment if relapse occurred within 6 months of completion. Prior radiation treatment (HNSCC) and previous concomitant or neoadjuvant chemotherapy completed more than 6 months before starting first-line therapy (NSCLC or HNSCC) were allowed. There were no further inclusion requirements regarding prior treatment for patients with NSCLC. Other key eligibility criteria are listed in the Supplementary Methods section (Supplementary Appendix).

Exclusion criteria included: prior exposure to an ATR inhibitor; unresolved toxicities (except alopecia) from prior therapy of National Cancer Institute Common Terminology Criteria for Adverse Events (NCI CTCAE) version 4.03 grade ≥2; spinal cord compression or brain metastases unless asymptomatic, stable and not requiring steroids for ≥4 weeks before starting study treatment; and inadequate organ (hepatic, renal, bone marrow, cardiac) function. Additional exclusion criteria are listed in the Supplementary Methods section (Supplementary Appendix).

All patients provided written informed consent to their participation in the study. The study protocol was approved by the institutional review board or ethics committee for each participating centre. The study was run in accordance with ethical principles originating in the Declaration of Helsinki and consistent with the International Conference on Harmonisation and Good Clinical Practice guidelines, and applicable regulatory requirements.

### Study design and treatment

The dose-escalation part explored increasing ceralasertib doses in combination with fixed-dose durvalumab (1500 mg IV every 4 weeks [Q4W]). Treatment comprised a 7- or 14-day lead-in period (Cycle 0) of ceralasertib monotherapy, followed by 28-day treatment cycles (Supplementary Fig. S1). The ceralasertib starting dose was 80 mg twice daily (BID), given orally for 14 days in Cycle 0 and on Days 22–28 from Cycle 1 onwards. In subsequent cohorts, ceralasertib doses were 160 mg BID, 320 mg once daily (QD) or 240 mg BID for 7 or 14 days (cohort-dependent) in Cycle 0 and for 7 days (Days 22–28) or 14 days (Days 15–28) from Cycle 1 onwards. Treatment breaks were designed to allow bone marrow recovery [30] and to replicate the intermittent dosing schedules potentially linked to improved antitumour activity reported in preclinical studies [29]. From Cycle 1 onwards, patients received durvalumab on Day 1 of each cycle.

Each dose-escalation cohort included at least 3 evaluable patients and dose escalation followed a ‘rolling six design’. The decision to escalate or reduce the ceralasertib dose in subsequent cohorts was based on assessment of safety and tolerability data collected up until the time of study drug administration on Cycle 2, Day 1 for these patients; i.e., following 7–14 days of ceralasertib dosing in Cycle 0, prior to durvalumab on Day 1, Cycle 1, and 7–14 days of ceralasertib dosing post-durvalumab in Cycle 1. Dose escalation rules and dose-limiting toxicities (DLTs) are defined in the Supplementary Methods (Supplementary Appendix). Ceralasertib doses and schedules were defined by a Safety Review Committee (SRC) and informed by the maximum tolerated dose of ceralasertib and emerging data from across the ceralasertib programme; the durvalumab dose was not changed. Ceralasertib dosing interruptions and reductions were permitted for clinically significant or unacceptable toxicity; no reduction in durvalumab dose was permitted.

In addition to the dose-escalation cohorts, patients could be recruited to a biopsy cohort (Supplementary Methods, Supplementary Appendix), in which pre-treatment and on-treatment tumour samples were collected before the first dose of ceralasertib (during screening) and 1–6 hours post-dose on Day 5 (±1 day) of Cycle 0. Patients in the biopsy cohort received ceralasertib monotherapy 240 mg BID for 7 days during Cycle 0 and 240 mg BID on Days 15–28 from Cycle 1 onwards, plus durvalumab 1500 mg IV Q4W from Cycle 1 Day 1 (Supplementary Fig. 1). This cohort aimed to characterise the pharmacodynamic effects of ATR inhibition by ceralasertib in the tumour.

In all cohorts, patients received ceralasertib and durvalumab as long as they continued to show clinical benefit, per investigator judgement, or until confirmed disease progression, unacceptable toxicity, or meeting another discontinuation criterion.

### Endpoints and assessments

The primary endpoint was the safety and tolerability of ceralasertib in combination with durvalumab in terms of adverse events (AEs), DLTs, laboratory data, vital signs, electrocardiogram changes, and physical examination. Secondary endpoints were ceralasertib pharmacokinetics and pharmacodynamics following a single dose, and at steady state after multiple dosing, in combination with durvalumab; tumour response per Response Evaluation Criteria for Solid Tumours (RECIST) v1.1, duration of response (DoR), progression-free survival (PFS) and overall survival (OS); and biomarkers of ceralasertib activity, including functional ATR inhibition. Exploratory analyses included additional pharmacodynamic biomarkers and biomarkers predictive of response or resistance to ceralasertib (to be reported elsewhere).

Patients were monitored regularly for safety throughout and until 90 days after the last dose of treatment; AEs were graded per NCI CTCAE v4.03. Tumours were assessed at baseline and every 8 weeks after starting combination treatment (Cycle 1, Day 1) until objective disease progression or consent withdrawal. Assessment frequency could be revised to every 16 weeks in patients treated with ceralasertib for >2 years with stable disease or better. All patients were followed for survival every 8 weeks after disease progression.

### Pharmacokinetics and pharmacodynamics

Blood samples for determination of ceralasertib pharmacokinetics were collected after a single dose of ceralasertib monotherapy (Cycle 0, Day 1), after multiple doses of ceralasertib monotherapy (Cycle 0, Day 5 or 8; both considered steady-state concentrations of ceralasertib), and after the last ceralasertib dose in Cycle 1 administered in combination with durvalumab (except in the biopsy cohort; Supplementary Fig. 2). No pharmacokinetic analysis was performed on the ceralasertib metabolite because of the low parent-to-metabolite ratio. Urine samples were collected to determine ceralasertib concentrations after a single dose of ceralasertib monotherapy and at steady state (Cycle 0, Day 1 and Day 8, respectively; except in the biopsy cohort).

In the biopsy cohort, biopsies were prepared as formalin-fixed paraffin-embedded (FFPE) tumour blocks and as frozen tumour samples (for multiplexed immuno-multiple reaction monitoring mass spectrometry – data not reported herein). FFPE tumour samples were analysed by immunohistochemistry (IHC) assay for ATM and pRAD50 (phosphorylated Ser635 on RAD50) biomarkers as previously described [35, 36]. Slides were scanned at x20 using an Aperio AT2 scanner (Leica). HALO image analysis software (Indica Labs) was used to quantify pRAD50 by determining median optical density using a cytonuclear algorithm and tumour tissue classifier. Classifier and algorithm training was supervised by a pathologist. Percentage of ATM-positive tumour cells was evaluated by a pathologist. For inclusion in the paired biopsy analysis of pRAD50, at least one biopsy core taken prior to treatment and one taken on treatment were required that met the following criteria: ≥100 tumour cells per pathologist review of a hematoxylin and eosin-stained slide; ATM staining intensity on IHC of 2+ in lymphocytes as a positive control; ≥10% ATM expression in at least one of the biopsies.

Peripheral blood samples were collected in acid citrate dextrose and Cyto-Chex® tubes. These were forwarded directly to Q2 Solutions for flow cytometry analysis to evaluate relative proportions of T cells, B cells and natural killer (NK) cells, and biomarkers associated with activation/proliferation of these cell types. Samples were collected at screening and pre-dose: on Days 1, 5 (biopsy cohort), 8, and 11 of Cycle 0; on Days 1, 8, 15 and 22 of Cycles 1 and 2; and on Days 1, 15, and 22 of Cycle 3 (Supplementary Fig. 2).

### Statistical analysis

The safety analysis set included all patients treated with at least one dose of ceralasertib; pharmacokinetic analyses included all dosed patients with reportable ceralasertib plasma concentrations and no key AEs or protocol deviations that might impact pharmacokinetics. Data were summarised using summary statistics. Time-to-event data were analysed using Kaplan–Meier methodology.

## Results

### Patients and treatment

Between January 2016 and April 2021, 78 patients were screened for enrolment in Module 3 at 8 sites in France, the Republic of Korea, the UK and the USA; 60 patients received ceralasertib treatment (of whom 58 also received durvalumab) across six dose-escalation cohorts (*n* = 37) and the biopsy cohort (*n* = 23) (Supplementary Figs.1). All 60 patients were evaluable for response and safety.

The median age across all cohorts was 62.0 years; 42 (70.0%) patients were male, 55 (91.7%) had metastatic disease, 37 (61.7%) had received ≥2 prior lines of chemotherapy, and 47 (78.3%) and 13 (21.7%) had NSCLC and HNSCC, respectively. The median number of prior chemotherapy regimens was 2; 50 (83.3%) and 20 (33.3%) of patients had received prior cytotoxic chemotherapy and immunotherapy, respectively, including 43/47 (91.5%) and 21/47 (44.7%) patients with NSCLC and 9/13 (69.2%) and 3/13 (23.1%) with HNSCC, respectively (Table 1). At data cutoff (Feb 21, 2022), 58 (96.7%) patients had discontinued ceralasertib; the most common reason was disease progression (*n* = 46; 76.7%), followed by AEs (*n* = 6; 10.0%), patient withdrawal (*n* = 4, 6.7%) and other reasons (*n* = 2, 3.3%). Two patients remained on treatment, one each from Cohort 1 (ceralasertib 80 mg BID) and the biopsy cohort (ceralasertib 240 mg BID). Fifty-six (93.3%) patients had discontinued durvalumab; the most common reason was disease progression (*n* = 44; 73.3%), followed by AEs (*n* = 6; 10.0%), patient withdrawal (*n* = 3, 5.0%) and other reasons (*n* = 3, 5.0%).

**Table 1:** Baseline patient demographics and disease characteristics

	Total (*N* = 60)	Cohort 1 (*n* = 9)	Cohort 2 (*n* = 3)	Cohort 3 (*n* = 3)	Cohort 4 (*n* = 7)	Cohort 5 (*n* = 7)	Cohort 6 (*n* = 8)	Biopsy cohort (*n* = 23)
Median age, years (range)	62.0 (33–81)	60.0 (33–71)	62.0 (56–66)	61.0 (53–69)	61.0 (50–68)	62.0 (55–67)	65.5 (51–75)	63.0 (41–81)
Age group, years								
<50	3 (5.0)	2 (22.2)	0	0	0	0	0	1 (4.3)
≥50 to <65	36 (60.0)	5 (55.6)	2 (66.7)	2 (66.7)	5 (71.4)	6 (85.7)	4 (50.0)	12 (52.2)
≥65	21 (35.0)	2 (22.2)	1 (33.3)	1 (33.3)	2 (28.6)	1 (14.3)	4 (50.0)	10 (43.5)
Sex, *n* (%)								
Male	42 (70.0)	8 (88.9)	3 (100.0)	3 (100.0)	4 (57.1)	4 (57.1)	4 (50.0)	16 (69.6)
Female	18 (30.0)	1 (11.1)	0	0	3 (42.9)	3 (42.9)	4 (50.0)	7 (30.4)
Race, *n* (%)								
White	36 (60.0)	7 (77.8)	2 (66.7)	2 (66.7)	3 (42.9)	1 (14.3)	8 (100.0)	13 (56.5)
Asian	20 (33.3)	2 (22.2)	1 (33.3)	1 (33.3)	3 (42.9)	4 (57.1)	0	9 (39.1)
Black or African American	2 (3.3)	0	0	0	0	1 (14.3)	0	1 (4.3)
Other	2 (3.3)	0	0	0	1 (14.3)	1 (14.3)	0	0
ECOG performance status, *n* (%)*								
0	11 (18.3)	3 (33.3)	1 (33.3)	0	0	0	1 (12.5)	6 (26.1)
1	47 (78.3)	6 (66.7)	2 (66.7)	3 (100.0)	7 (100.0)	7 (100.0)	7 (87.5)	15 (65.2)
Overall disease classification, *n* (%)								
Locally advanced	5 (8.3)	1 (11.1)	0	0	1 (14.3)	0	0	3 (13.0)
Metastatic	55 (91.7)	8 (88.9)	3 (100.0)	3 (100.0)	6 (85.7)	7 (100.0)	8 (100.0)	20 (87.0)
Number of metastatic sites, *n* (%)^†^								
1	22 (36.7)	2 (22.2)	1 (33.3)	2 (66.7)	3 (42.9)	2 (28.6)	3 (37.5)	9 (39.1)
2	16 (26.7)	3 (33.3)	1 (33.3)	0	1 (14.3)	2 (28.6)	1 (12.5)	8 (34.8)
≥3	17 (28.3)	3 (33.3)	1 (33.3)	1 (33.3)	2 (28.6)	3 (42.9)	4 (50.0)	3 (13.0)
Primary tumour location, *n* (%)								
Lung	47 (78.3)	4 (44.4)	1 (33.3)	3 (100.0)	7 (100.0)	7 (100.0)	8 (100.0)	17 (73.9)
Head and neck (inc. nasopharynx, larynx, trachea)	9 (15.0)	2 (22.2)	2 (66.7)	0	0	0	0	5 (21.7)
Oropharynx	2 (3.3)	2 (22.2)	0	0	0	0	0	0
Hypopharynx	1 (1.7)	0	0	0	0	0	0	1 (4.3)
Oral cavity	1 (1.7)	1 (11.1)	0	0	0	0	0	0
Histology, *n* (%)^‡^								
Adenocarcinoma	31 (51.7)	4 (44.4)	0	2 (66.7)	5 (71.4)	5 (71.4)	8 (100)	7 (30.4)
Squamous cell	24 (40.0)	5 (55.6)	2 (66.7)	1 (33.3)	1 (14.3)	2 (28.6)	0	13 (56.5)
Non-small cell carcinoma	5 (8.3)	0	1 (33.3)	0	1 (14.3)	0	0	3 (13.0)
Time from diagnosis to first dose of ceralasertib, *n* (%)								
≤24 months	17 (28.3)	3 (33.3)	0	0	3 (42.9)	2 (28.6)	2 (25.0)	7 (30.4)
>24 months	43 (71.7)	6 (66.7)	3 (100.0)	3 (100.0)	4 (57.1)	5 (71.4)	6 (75.0)	16 (69.6)
Previous treatment modalities, n (%)								
Radiotherapy	32 (53.3)	7 (77.8)	3 (100.0)	3 (100.0)	1 (14.3)	4 (57.1)	2 (25.0)	12 (52.2)
Cytotoxic chemotherapy	50 (83.3)	8 (88.9)	3 (100.0)	2 (66.7)	4 (57.1)	6 (85.7)	8 (100.0)	19 (82.6)
Immunotherapy	20 (33.3)	0	1 (33.3)	0	2 (28.6)	1 (14.3)	1 (12.5)	15 (65.2)
Systemic therapy (not specified)	20 (33.3)	2 (22.2)	1 (33.3)	2 (66.7)	2 (28.6)	3 (42.9)	3 (37.5)	7 (30.4)
Hormonal therapy	1 (1.7)	0	0	0	0	0	1 (12.5)	0
Other	7 (11.7)	0	1 (33.3)	1 (33.3)	1 (14.3)	1 (14.3)	2 (25.0)	1 (4.3)
Number of prior lines of chemotherapy, *n* (%)								
1	13 (21.7)	2 (22.2)	0	1 (33.3)	4 (57.1)	1 (14.3)	1 (12.5)	4 (17.4)
2	13 (21.7)	1 (11.1)	0	1 (33.3)	2 (28.6)	2 (28.6)	2 (25.0)	5 (21.7)
≥3	24 (40.0)	6 (66.7)	3 (100.0)	1 (33.3)	1 (14.3)	4 (57.1)	4 (50.0)	5 (21.7)
Unknown	10 (16.7)	0	0	0	0	0	1 (12.5)	9 (39.1)^#^

Patients received ceralasertib at doses of 80 mg BID (Cohort 1), 160 mg BID (Cohort 2), 320 mg QD (Cohorts 3 and 4), and 240 mg BID (Cohorts 5 and 6, and the biopsy cohort) in combination with a fixed dose of durvalumab (1500 mg IV Q4W); the number of dosing days per cycle varied between cohorts receiving the same daily dose of ceralasertib (see Supplementary Figs.1).

*Performance status is missing for two patients from the biopsy cohort.

^†^In patients with metastatic disease only; percentages are based on the total number of patients treated per cohort.

^‡^Adenocarcinoma includes the terms adenocarcinoma (*n* = 12), adenocarcinoma not otherwise specified (*n* = 12), adenocarcinoma typical (*n* = 3), adenocarcinoma diffuse type (*n* = 1), adenocarcinoma: acinar (*n* = 1), non-mucinous adenocarcinoma of right middle lobe (*n* = 1), and papillary adenocarcinoma (*n* = 1). Squamous cell carcinoma includes the terms basaloid squamous cell carcinoma (*n* = 1), moderately differentiated keratinising squamous cell carcinoma (*n* = 1), non-small-cell lung cancer (squamous) (*n* = 1), squamous cell carcinoma (*n* = 18), and squamous cell carcinoma not otherwise specified (*n* = 3). Non-small-cell carcinoma includes the terms non-small-cell carcinoma (*n* = 1), non-small-cell lung carcinoma (*n* = 2), NSCLC (*n* = 1), and poorly differentiated non-small-cell carcinoma (*n* = 1).

^#^High rate of unknown prior lines of chemotherapy in the biopsy data due to missing data.

*BID* twice daily, *ECOG* Eastern Cooperative Oncology Group, *IV* intravenous, *Q4W*, every 4 weeks, *QD* once daily.

Across all cohorts, median total duration (first to last dose) of ceralasertib treatment was 4.4 months (range, 0.1–70.4) and of durvalumab was 3.8 months (range, <0.1–70.0). In Cycle 0 and in Cycle 1 and beyond, respectively, 3 (5.0%) and 13 (21.7%) patients had ceralasertib dosing interruptions and 0 and 11 (18.3%) had ceralasertib dose reductions.

### Safety

#### Treatment-emergent adverse events (TEAEs)

Across all cohorts, 59 (98.3%) patients had TEAEs, including 31 (51.7%) who had Grade ≥3 TEAEs (Table 2; Supplementary Table S1). The most common Grade ≥3 TEAEs by recorded Medical Dictionary for Regulatory Activities (MedDRA) preferred term were thrombocytopenia/platelet count decreased (pooled terms, *n* = 11; 18.3%), anaemia (*n* = 9; 15.0%), and lower respiratory tract infection (*n* = 4; 6.7%). Overall, 48 (80.0%) patients had treatment-related AEs (TRAEs), including 18 (30.0%) who had Grade ≥3 TRAEs (Supplementary Table S1). Forty-six (76.7%) and 27 (45.0%) patients had TRAEs specifically related to ceralasertib or durvalumab only, respectively, including 12 (20.0%) and 2 (3.3%) who had Grade ≥3 events. The most common ceralasertib-related TRAEs of any Grade were nausea (*n* = 26, 43.3%), anaemia (*n* = 21; 35.0%), thrombocytopenia/platelet count decreased (*n* = 19, 31.7%), fatigue (*n* = 18, 30.0%), vomiting (*n* = 14, 23.3%), and decreased appetite (*n* = 13, 21.7%).

**Table 2:** TEAEs occurring at any Grade in ≥15% or at Grade ≥3 in >5% of patients across all cohorts (safety analysis set)

TEAE, *n* (%)	Total (*N* = 60)	Cohort 1 (*n* = 9)	Cohort 2 (*n* = 3)	Cohort 3 (*n* = 3)	Cohort 4 (*n* = 7)	Cohort 5 (*n* = 7)	Cohort 6 (*n* = 8)	Biopsy cohort (n = 23)	Cohort 6 + Biopsy cohort* (*n* = 31)
**All-grade TEAEs occurring in** ≥ **15% of patients overall**									
Any	59 (98.3)	9 (100.0)	3 (100.0)	3 (100.0)	7 (100.0)	7 (100.0)	8 (100.0)	22 (95.7)	30 (96.8)
Nausea	35 (58.3)	6 (66.7)	1 (33.3)	3 (100.0)	3 (42.9)	4 (57.1)	7 (87.5)	11 (47.8)	18 (58.1)
Anaemia	34 (56.7)	5 (55.6)	1 (33.3)	2 (66.7)	4 (57.1)	2 (28.6)	7 (87.5)	13 (56.5)	20 (64.5)
Fatigue	32 (53.3)	4 (44.4)	3 (100.0)	3 (100.0)	5 (71.4)	1 (14.3)	6 (75.0)	10 (43.5)	16 (51.6)
Vomiting	21 (35.0)	3 (33.3)	1 (33.3)	1 (33.3)	4 (57.1)	1 (14.3)	5 (62.5)	6 (26.1)	11 (35.5)
Thrombocytopenia/platelet count decreased (pooled terms)	20 (33.3)	1 (11.1)	2 (66.7)	0	3 (42.9)	1 (14.3)	3 (37.5)	10 (43.5)	13 (41.9)
Decreased appetite	19 (31.7)	3 (33.3)	1 (33.3)	1 (33.3)	3 (42.9)	3 (42.9)	1 (12.5)	7 (30.4)	8 (25.8)
Diarrhoea	17 (28.3)	2 (22.2)	0	2 (66.7)	3 (42.9)	0	2 (25.0)	8 (34.8)	10 (32.3)
Cough	15 (25.0)	6 (66.7)	1 (33.3)	1 (33.3)	3 (42.9)	2 (28.6)	0	2 (8.7)	2 (6.5)
LRTI	14 (23.3)	2 (22.2)	1 (33.3)	0	1 (14.3)	0	5 (62.5)	5 (21.7)	10 (32.3)
Dizziness	13 (21.7)	3 (33.3)	0	1 (33.3)	3 (42.9)	0	0	6 (26.1)	6 (19.4)
Dyspnoea	12 (20.0)	3 (33.3)	0	1 (33.3)	1 (14.3)	1 (14.3)	3 (37.5)	3 (13.0)	6 (19.4)
Pruritus	12 (20.0)	2 (22.2)	1 (33.3)	1 (33.3)	2 (28.6)	1 (14.3)	2 (25.0)	3 (13.0)	5 (16.1)
Constipation	11 (18.3)	2 (22.2)	0	1 (33.3)	1 (14.3)	2 (28.6)	3 (37.5)	2 (8.7)	5 (16.1)
Headache	11 (18.3)	1 (11.1)	1 (33.3)	1 (33.3)	2 (28.6)	1 (14.3)	0	5 (21.7)	5 (16.1)
Insomnia	11 (18.3)	2 (22.2)	1 (33.3)	0	1 (14.3)	0	4 (50.0)	3 (13.0)	7 (22.6)
Rash	11 (18.3)	1 (11.1)	0	1 (33.3)	4 (57.1)	0	2 (25.0)	3 (13.0)	5 (16.1)
Pyrexia	10 (16.7)	1 (11.1)	1 (33.3)	0	0	1 (14.3)	5 (62.5)	2 (8.7)	7 (22.6)
Arthralgia	9 (15.0)	2 (22.2)	0	0	1 (14.3)	1 (14.3)	2 (25.0)	3 (13.0)	5 (16.1)
Dry skin	9 (15.0)	2 (22.2)	0	1 (33.3)	2 (28.6)	0	2 (25.0)	2 (8.7)	4 (12.9)
URTI	9 (15.0)	1 (11.1)	1 (33.3)	0	1 (14.3)	0	1 (12.5)	5 (21.7)	6 (19.4)
UTI	9 (15.0)	1 (11.1)	0	2 (66.7)	2 (28.6)	0	1 (12.5)	3 (13.0)	4 (12.9)
WBC count decreased	9 (15.0)	1 (11.1)	1 (33.3)	0	1 (14.3)	0	1 (12.5)	5 (21.7)	6 (19.4)
**Grade** ≥ **3 TEAEs occurring in** ≥ **5% of patients overall**									
Thrombocytopenia/platelet count decreased (pooled terms)	11 (18.3)	0	1 (33.3)	0	1 (14.3)	0	2 (25.0)	7 (30.4)	9 (29.0)
Anaemia	9 (15.0)	0	0	0	2 (28.6)	0	2 (25.0)	5 (21.7)	7 (22.6)
LRTI	4 (6.7)	2 (22.2)	0	0	0	0	1 (12.5)	1 (4.3)	2 (6.5)
Neutropenia	3 (5.0)	0	0	0	0	0	1 (12.5)	2 (8.7)	3 (9.7)
Pneumonia	3 (5.0)	1 (11.1)	0	0	0	0	2 (25.0)	0	2 (6.5)

Ceralasertib doses (Cycle 1 onwards): 80 mg BID, 7 days (Cohort 1); 160 mg BID, 7 days (Cohort 2); 320 mg QD, 7 days (Cohort 3) or 14 days (Cohort 4); 240 mg BID, 7 days (Cohort 5) or 14 days (Cohort 6 and biopsy cohort). Fixed-dose durvalumab: 1500 mg IV Q4W. See Supplementary Fig. 1.

TEAEs are shown in descending order of incidence overall. TEAEs are AEs with an onset date on or after the date of the first dose of study drug (ceralasertib or durvalumab), up to and including 90 days following the date of the last dose of study drug. *RP2D: data pooled from Cohort 6 and biopsy cohort.

*AE* adverse event, *BID* twice daily, *IV* intravenous, *LRTI* lower respiratory tract infection, *Q4W* every 4 weeks, *QD* once daily, *TEAE* treatment-emergent adverse event, *URTI* upper respiratory tract infection, *UTI* urinary tract infection, *WBC* white blood cell.

Across all cohorts, 23 (38.3%) patients had serious AEs (SAEs) (Supplementary Table S2), including 7 (11.7%) who had treatment-related SAEs (Supplementary Table S1). Three (5.0%) and 3 (5.0%) patients, respectively, had SAEs specifically related to ceralasertib or durvalumab. Four (6.7%) patients had AEs that led to death: haemoptysis in 1 patient in Cohort 5 (ceralasertib 240 mg BID), pneumonia in 1 patient in Cohort 6 (ceralasertib 240 mg BID), and cardiorespiratory arrest and pulmonary haemorrhage each in 1 patient in the biopsy cohort (ceralasertib 240 mg BID); none were considered causally related to treatment.

Seven (11.7%) patients had TEAEs that led to discontinuation of ceralasertib (Supplementary Table S1), which were SAEs in 5 (8.3%) patients. One patient in Cohort 2 (ceralasertib 160 mg BID) discontinued due to Grade 2 musculoskeletal pain that was considered immune-mediated and related to both ceralasertib and durvalumab. One patient in Cohort 5 (ceralasertib 240 mg BID) discontinued due to an SAE of Grade 3 durvalumab-related Guillain-Barré syndrome. One patient in Cohort 6 (ceralasertib 240 mg BID) discontinued due to an SAE of Grade 2 durvalumab-related immune-mediated neuropathy. Four patients in the biopsy cohort (ceralasertib 240 mg BID) discontinued due to an SAE of Grade 3 durvalumab-related pneumonitis, Grade 2 syncope (unrelated), and the Grade 5 TEAEs of cardiorespiratory arrest and pulmonary haemorrhage (unrelated).

#### Dose-limiting toxicities

Two patients had DLTs. One patient in Cohort 4 (ceralasertib 320 mg QD, 14 days per cycle) had Grade 3 thrombocytopenia/platelet count decreased and Grade 3 anaemia during Cycle 0; while this myelosuppression did not meet DLT criteria per the protocol (see Supplementary Appendix, Methods section), the SRC agreed with the investigator’s suggestion to reduce this patient’s dose to ceralasertib 320 mg QD for 7 days per cycle and classified the myelosuppression as a DLT. A second patient had a DLT of Grade 4 thrombocytopenia/platelet count decreased with Grade 3 neutropenia accompanied by systemic chest infection in Cohort 6 (ceralasertib 240 mg BID, 14 days per cycle).

#### Recommended Phase 2 dose (RP2D)

Ceralasertib 240 mg BID, Days 15–28, plus durvalumab 1500 mg, Day 1, in 28-day cycles, was identified as the RP2D based on assessment of aggregated safety data. Of 31 patients treated at the RP2D (pooled from Cohort 6 and the biopsy cohort), 30 (96.8%) had TEAEs, including 20 (64.5%) who had Grade ≥3 TEAEs (Table 2). The most common Grade ≥3 TEAEs at the RP2D by recorded MedDRA preferred term were thrombocytopenia/platelet count decreased (pooled terms, *n* = 9; 29.0%), anaemia (*n* = 7; 22.6%), neutropenia (*n* = 3, 9.7%), and pneumonia, lower respiratory tract infection, and atrial fibrillation (each *n* = 2; 6.5%). Platelet and neutrophil count data indicated that a 2-week off-treatment period was sufficient for Grade ≥3 thrombocytopenia/platelet count decreased and neutropenia to recover before starting ceralasertib treatment in the next cycle (Supplementary Fig. 3).

### Efficacy

**Fig. 1:**
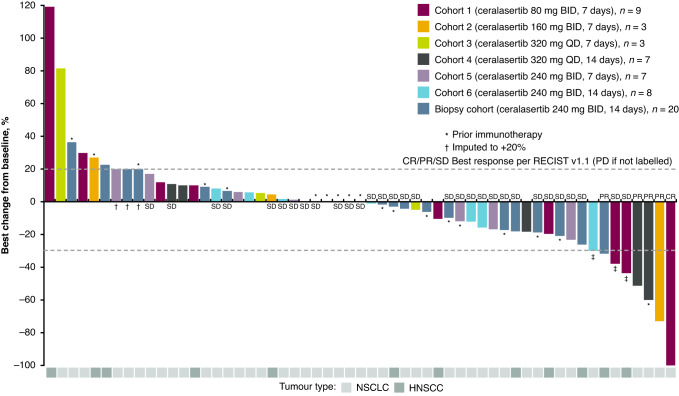
Best percentage change from baseline in target lesion size. Three patients with incomplete post-baseline tumour assessments were not evaluable for tumour response and were excluded from the plot. *Received prior immunotherapy. ^†^Best percentage change from baseline before disease progression and any subsequent cancer therapy was imputed to 20 for patients with missing target lesion data who died or progressed. ^‡^The patient with NSCLC in Cohort 6, with a best change from baseline of –30% in target lesions, had a best response of PD per RECIST v1.1 based on assessment of non-target lesions. The patient with NSCLC in Cohort 1 with a best change from baseline of –38% in target lesions, had a best response of SD per RECIST v1.1 based on an earlier response assessment, with subsequent PD based on assessment of non-target lesions and occurrence of new lesions at the time of the best change from baseline in target lesions. The patient with HNSCC in Cohort 1 with a best change from baseline of –44% in target lesions had a best response of SD per RECIST v1.1 because no confirmation assessment was performed in order to confirm a response of PR. BID, twice daily; CR, complete response; HNSCC head and neck squamous cell carcinoma (including oropharyngeal, hypopharyngeal, and buccal cancer), NSCLC non-small-cell lung cancer, PD progressive disease, PR partial response, QD once daily, RECIST Response Evaluation Criteria In Solid Tumours, SD stable disease.

Best percentage change from baseline in target lesion size across cohorts is shown in Fig. 1. Overall, 5/60 patients had an objective response; the objective response rate (ORR) was 8.3% (80% confidence interval [CI], 4.1–14.9) (Table 3). Median DoR was 105.4 (range, 16.7–277.1) weeks (Table 3; Supplementary Figs.4). One complete response occurred in a 59-year-old male patient with Stage IV NSCLC in Cohort 1 (ceralasertib 80 mg BID for 7 days) who had received 3 prior regimens of chemotherapy; the DoR was 63.7+ months (5.3+ years; treatment and response were ongoing at data cutoff). Four partial responses occurred in 1 patient with NSCLC in Cohort 2 (ceralasertib 160 mg BID; DoR 29.7 months, ongoing), 2 patients with NSCLC in Cohort 4 (ceralasertib 320 mg QD; DoRs 20.2 and 24.2 months), and 1 patient with HNSCC in the biopsy cohort (RP2D, ceralasertib 240 mg BID; DoR 3.8 months). One responder with NSCLC in Cohort 4 had been previously treated with pembrolizumab (treatment duration 46 days; best objective response, progressive disease); all other responders were immunotherapy-naïve.

**Table 3:** Summary of confirmed tumour response and response duration

Response, *n* (%)	Total (*N* = 60)	Cohort 1 (*n* = 9)	Cohort 2 (*n* = 3)	Cohort 3 (*n* = 3)	Cohort 4 (*n* = 7)	Cohort 5 (*n* = 7)	Cohort 6 (*n* = 8)	Biopsy cohort (*n* = 23)	Cohort 6 + Biopsy cohort* (*n* = 31)
Objective response (rate), *n* (%)	5 (8.3)	1 (11.1)	1 (33.3)	0	2 (28.6)	0	0	1 (4.3)	1 (3.2)
[80% CI for response rate]	[4.11–14.91]	[1.16–36.84]	[3.45–80.42]	[0–53.58]	[7.88–59.62]	[0–28.03]	[0–25.01]	[0.46–15.88]	[0.34–11.98]
Best objective response, *n* (%)^†^									
Complete response	1 (1.7)	1 (11.1)	0	0	0	0	0	0	0
Partial response	4 (6.7)	0	1 (33.3)	0	2 (28.6)	0	0	1 (4.3)	1 (3.2)
Stable disease ≥35 days^‡^	31 (51.7)	3 (33.3)	1 (33.3)	1 (33.3)	3 (42.9)	5 (71.4)	5 (62.5)	13 (56.5)	18 (58.1)
Progressive disease^#^	21 (35.0)	5 (55.6)	1 (33.3)	2 (66.7)	2 (28.6)	2 (28.6)	3 (37.5)	6 (26.1)	9 (29.0)
Not evaluable^¶^	3 (5.0)	0	0	0	0	0	0	3 (13.0)	3 (9.7)
DoR, weeks^§^	105.4 (16.7–277.1)	277.1+	129.1+	NA	87.7, 105.4	NA	NA	16.7	16.7
12-month DoR rate, %	80.0	100	100	NA	100	NA	NA	0	0

Ceralasertib doses (Cycle 1 onwards): 80 mg BID, 7 days (Cohort 1); 160 mg BID, 7 days (Cohort 2); 320 mg QD, 7 days (Cohort 3) or 14 days (Cohort 4); 240 mg BID, 7 days (Cohort 5) or 14 days (Cohort 6 and biopsy cohort). Fixed-dose durvalumab: 1500 mg IV Q4W. See Supplementary Figs.1.

*RP2D: data pooled from Cohort 6 and biopsy cohort.

^†^One of the responders in Cohort 4 (a patient with NSCLC) had been previously treated with pembrolizumab; all other responders were immunotherapy-naïve.

^‡^Includes 1 patient in Cohort 1 with an unconfirmed complete or partial response.

^#^Includes patients with RECIST-defined progression (*n* = 18) and patients who died (*n* = 3; in 2 patients the primary cause of death was from disease, and in 1 patient the primary cause of death was an SAE of cardiopulmonary arrest; none of the deaths were deemed related to treatment or study procedure).

^¶^Incomplete post-baseline assessments.

^§^Responses ongoing at time of data cutoff indicated by +. Kaplan–Meier median and range shown in the ‘Total’ column.

*BID* twice daily, *CI* confidence interval, *DoR* duration of response, *IV* intravenous, *NA* not applicable, *NR* not reached, *NSCLC* non-small-cell lung cancer, *Q4W* every 4 weeks, *QD* once daily, *RECIST* Response Evaluation Criteria in Solid Tumours, *RP2D* recommended Phase 2 dose, *SAE* serious adverse event.

**Fig. 2:**
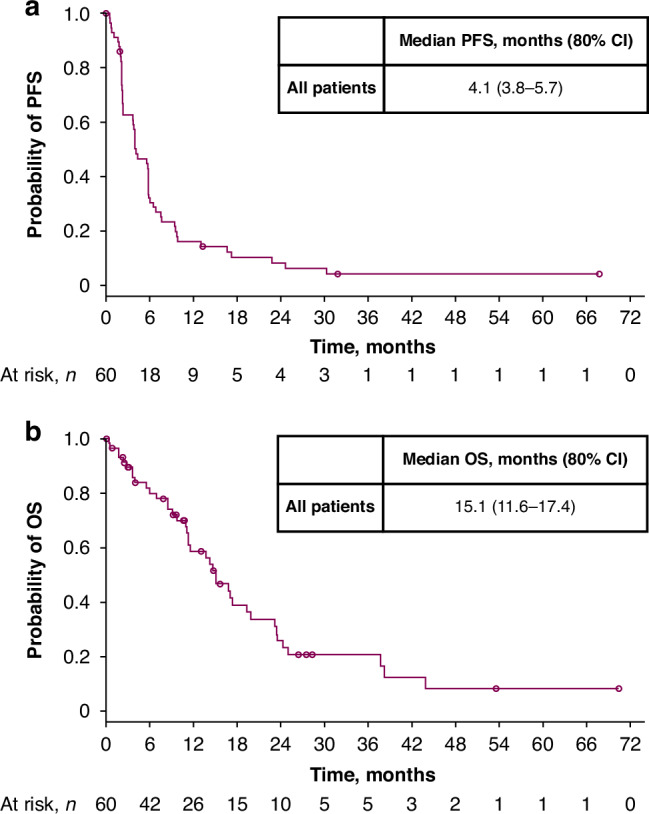
PFS and OS. Kaplan–Meier analyses of PFS (**a**) and OS (**b**) in the overall study population. CI confidence interval, OS overall survival, PFS progression-free survival.

Across cohorts, median PFS was 4.1 months (80% CI, 3.8–5.7) and median OS was 15.1 months (80% CI, 11.6–17.4); 12-month PFS and OS rates were 16.1% (80% CI, 10.4–22.9) and 58.6% (80% CI, 49.0–67.0), respectively (Fig. 2). In patients with NSCLC, median PFS was 5.6 months (80% CI, 3.8–5.8); median OS was 15.1 months (80% CI, 13.7–19.3). In patients with HNSCC, median PFS was 3.8 months (80% CI, 2.3–5.8); median OS was 11.1 months (80% CI, 9.7–24.3).

### Pharmacokinetics

**Fig. 3:**
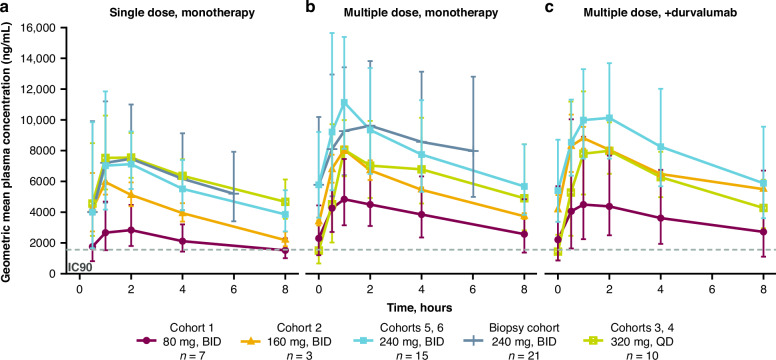
Ceralasertib plasma concentration versus time profi les. Geometric mean (±GSD) of ceralasertib plasma concentrations versus time profiles following **a** single-dose monotherapy; **b** multiple-dose monotherapy; and **c** multiple dose in combination with durvalumab. 160 mg BID multiple dose plots have *n* = 2. BID twice daily, GSD geometric standard deviation, QD once daily, IC_9_, ATR target inhibition cell IC_90_ value, defined as the concentration of ceralasertib required to inhibit ATR-dependent phosphorylation of CHK1 Serine345 by 90% in HT-29 cells by immunofluorescence imaging assay, as described in [7].

Overall, 58 patients provided samples for pharmacokinetic analysis. Geometric mean plasma concentration–time profiles (Fig. 3) showed that ceralasertib exposure over 8 hours was above the ATR target inhibition IC_90_ value [7] at all dose levels. Single-dose and steady-state pharmacokinetic parameters with ceralasertib monotherapy are summarised in Supplementary Table S3. Ceralasertib was rapidly absorbed into plasma (median t_max_ 1.01–1.51 hours across doses). Geometric mean accumulation ratios with ceralasertib 80 and 240 mg BID monotherapy, based on AUC_0–6_, were 2.07 and 1.44, respectively. Single-dose and steady-state ceralasertib exposure appeared to increase in a less-than-dose-proportional manner; dose-normalised C_max_ and AUC_0–8_ were 1.15- and 1.3-fold higher, respectively, following a single dose of 80 mg compared with 240 mg (Supplementary Table S3). Data suggested that only a minimal increase in systemic exposure could be achieved at doses above 240 mg; steady-state systemic exposure was highest with ceralasertib 240 mg BID. Pharmacokinetic parameters were similar for ceralasertib in combination with durvalumab (Fig. 3, Supplementary Table S4), indicating a lack of drug-drug interaction. Excretion of ceralasertib in urine was low irrespective of dose or dosing frequency (arithmetic mean percentage excreted over 8 hours: 7.04–8.54% after a single dose, 10.7–14.3% at steady state).

### Pharmacodynamics in paired tumour biopsies

pRAD50 has been previously described as a potential pharmacodynamic biomarker of ATR inhibition; its increase is associated with DNA damage and mediated by ATM. Inhibition of ATR leads to an increase in DNA damage and induction of ATM activity, and ceralasertib treatment leads to an increase in pRAD50 in ATM-proficient preclinical models [7, 35]. Of 22 patients in the biopsy cohort, 14 had paired pre-treatment and on-treatment biopsies considered evaluable for pRAD50 IHC analysis. Of the non-evaluable paired biopsies (*n* = 8), 6 were non-evaluable due to insufficient tumour cells at one of the two timepoints, and 2 failed the ATM IHC internal quality control (2+ staining of lymphocytes) at one or both timepoints. An increase in pRAD50 was observed in 10/14 paired biopsies, with 7/10 having an over 2-fold increase in pRAD50, suggesting activation of DDR due to ATR inhibition and indicating target engagement and downstream biological activity in a proportion of these biopsies. (Supplementary Fig. 5).

### Pharmacodynamics in blood

Recent data suggest that ATR inhibition may have a potentially broad and profound impact on the TIME, promoting T-cell activity and influencing the number and function of myeloid cells [20, 29]. In peripheral blood samples, levels of monocytes and proliferating CD8+ T cells (CD8+ Ki67+ cells) were reduced during ceralasertib treatment; monocyte levels decreased in a dose-dependent manner. During the period when ceralasertib was not administered, monocytes and proliferating CD8+ T cells increased and returned to baseline (Supplementary Figs. 6, S7). With 14-day ceralasertib dosing in Cycle 0, the most pronounced decrease in monocytes was seen at the 240 mg BID dose level (Supplementary Fig. 6A). In contrast to monocyte modulation, there was no obvious dose-response relationship on decline or rebound effects in proliferating CD8+ T cells (Supplementary Fig. 6B), which were observed across all dose levels. The effects of ceralasertib on total T cell levels were less pronounced, suggesting treatment mostly affects proliferating T cells (data not shown), in line with the role of ATR in the replication stress response. When comparing 7-day and 14-day dosing of ceralasertib 240 mg BID monotherapy in Cycle 0, recovery of monocytes and CD8+ T cells appeared faster on a 7-day compared to a 14-day schedule (Supplementary Figs. 6C and 6D).

## Discussion

Escalating doses of ceralasertib in combination with fixed-dose durvalumab had a manageable safety profile in patients with previously treated, locally advanced/metastatic NSCLC or HNSCC. Across the dose-escalation cohorts, 2 patients experienced DLTs; there were no treatment-related deaths, and 11.7% of patients discontinued due to TEAEs. From Cycle 1 onwards, 21.7% of patients had ceralasertib dosing interruptions and 18.3% had dose reductions to manage toxicity. The safety profile was consistent with those already known for durvalumab and emerging (across a range of solid and haematological malignancies) for ceralasertib, with no new safety signals [20, 37–42]. The most common Grade ≥3 TEAEs were haematological; however, platelet and neutrophil count data indicated that 2 weeks off ceralasertib was sufficient for recovery of Grade ≥3 thrombocytopenia/platelet count decreases and neutropenia before treatment during the next cycle. While response rates were limited in patients with NSCLC or HNSCC, the responses in NSCLC were durable, with all patients remaining in response at 12 months; however, it should be acknowledged that three patients with NSCLC who responded were immunotherapy-naïve and so this efficacy may have been due to durvalumab. Additionally, our findings lack predictive biomarkers for these durable responses; further research is warranted in this regard.

Consistent with the role of ATR in the replication stress response, ATR inhibition leads to a downstream increase in DDR signalling and activation of ATM, which in turn induces phosphorylation of downstream targets, including pRAD50 [35]. In the current study, an increase in pRAD50 observed in 10/14 paired pre-treatment and on-treatment tumour biopsies collected from patients receiving ceralasertib 240 mg BID suggests target engagement in the tumour and subsequent increase in DNA damage.

In addition to the direct effects of ceralasertib on tumour cells through exacerbation of replication stress and DNA damage, evidence indicates that intermittent doses of ceralasertib can modulate the TIME [20, 29]. Intermittent on-off ceralasertib treatment in mouse syngeneic tumour models drove antitumour activity in a CD8+ T cell- and type-I interferon-dependent manner, with ceralasertib decreasing the number of tumour T cells displaying markers of exhaustion and myeloid-derived suppressor cells during the on-treatment Phase, enabling repopulation of the TIME with T cells that have improved function during the subsequent off-treatment period, and thus making the cells potentially more susceptible to the effects of anti-PD-L1 treatment [29]. Ceralasertib monotherapy was also found to modulate immune response in the first-in-human Phase 1 PATRIOT study in advanced solid tumours, with responding patients shown to have a more favourable CD8/Treg ratio, activation of NK cells, increased frequencies of effector memory CD4+ T cells, and modulation of cytokines and circulating myeloid-derived suppressor cells, as well as increases in tumour-infiltrating lymphocytes and inflammatory gene expression, compared with non-responding patients [20].

The current study also found clinical evidence of the immunomodulatory effects of intermittent ceralasertib dosing in analyses of peripheral blood samples; however, intratumoural analyses were not performed to determine whether these effects were reflected in the TIME. Ceralasertib had a pronounced effect on the numbers of circulating proliferating CD8+ T cells, with initial transient reductions followed by recovery to baseline levels in the ceralasertib off-treatment period, supporting intermittent dosing schedules of ceralasertib. The effects of ceralasertib on total T cell levels were less pronounced compared with proliferating T cells, likely due to proliferating T cells exhibiting increased replication stress and sensitivity to ATR inhibition [29].

Monocyte numbers were also reduced on ceralasertib treatment before returning to baseline once treatment was stopped; the most pronounced decrease was seen at the ceralasertib 240 mg BID dose. Although CD8+ T cells displayed the same longitudinal pattern as monocytes on ceralasertib treatment, there was a dose relationship with monocytes but not with levels of proliferating CD8+ T-cells, most likely due to higher noise and variability in the T-cell data. When comparing 7-day and 14-day dosing of ceralasertib 240 mg BID monotherapy in Cycle 0, there was an earlier recovery of monocytes and CD8+ T cells with 7-day dosing, which suggests this schedule might be more tolerable long-term. Indeed, a faster recovery of cells due to a longer off-treatment period might provide more time for repopulating the TIME with naïve or activated effector T cells.

Ceralasertib exposure did not increase in a dose-proportional manner following a single dose or at steady state, with minimal increases predicted above a dose of 240 mg BID. For this reason, and given the neutrophil and platelet recovery observed after a 2-week off-treatment period, the target engagement observed in the tumour, and the tolerability of this dose in combination with durvalumab, ceralasertib 240 mg BID on Days 15–28 plus durvalumab 1500 mg on Day 1 of each 28-day cycle, was identified as the RP2D. This treatment combination has also shown encouraging antitumour activity and manageable toxicity in Phase 2 trials in patients with previously treated advanced gastric cancer [23], metastatic melanoma after failure of anti-PD-1 treatment [21] and NSCLC after failure of anti-PD-(L)1 treatment [22].

Expansion cohorts for ceralasertib in combination with durvalumab were planned in Module 3 but did not start because, following the results of the dose-escalation cohorts, Cohort 9 of the Phase 2 HUDSON study (NCT03334617) was initiated in patients with metastatic NSCLC previously treated with a platinum-doublet regimen and with progression on anti-PD-(L)1-containing therapy. HUDSON is an umbrella study evaluating the efficacy and safety of novel agents in combination with durvalumab in biomarker-matched and biomarker non-matched patients. HUDSON evaluated ceralasertib 240 mg BID on a 7-day dosing schedule [22], aligning the dosing schedule across the ceralasertib programme in NSCLC and melanoma (MONETTE study) [43]. For this combination, the ORR was 13.9% (*n* = 79), the median PFS was 5.8 months, and the median OS was 17.4 months, which were all superior to any of the other combinations evaluated in HUDSON (durvalumab plus olaparib, danvatirsen, or oleclumab). Particularly promising results were observed in ATM-deficient patients (*n* = 23), with an ORR of 26.1% with the ceralasertib plus durvalumab combination [22]. These findings from HUDSON, as well as the median PFS (5.6 months) and OS (15.1 months) in the present study in patients with NSCLC, appear promising in the context of median PFS of 2.8–4.5 months and median OS of 8.1–11.3 months with docetaxel following platinum-based chemotherapy, with or without immunotherapy [44–51].

More broadly, the results from HUDSON and the present study indicate the potential for combination regimens comprising immunotherapy plus DDR pathway inhibitors in advanced NSCLC, as reviewed recently [52]. As of June 2025, several other ATR inhibitors are currently in Phase 1/2 clinical trials in patients with advanced solid tumours, although no clinical data in combination with immunotherapy have yet been reported [53]. Berzosertib (M6620/VX-970) has been shown to be well tolerated with preliminary efficacy in patients with advanced solid tumours, both as monotherapy and in combination with carboplatin, topotecan, gemcitabine, or radiation [54–57]; an ongoing Phase 1b/2 study is evaluating berzosertib in combination with carboplatin/gemcitabine/pembrolizumab in patients with chemotherapy-naïve advanced squamous NSCLC (NCT04216316). Elimusertib (BAY1895344) monotherapy has been shown to be well tolerated with preliminary activity in patients with advanced solid tumours and DDR deficiency [58, 59]. The combination of elimusertib, pembrolizumab, and stereotactic body radiation therapy is currently being tested in a Phase 1 clinical trial for patients with unresectable or metastatic HNSCC within a previously irradiated field (NCT04576091) [34, 60]. Similar to ceralasertib, the clinical success of these agents, particularly in combination with chemotherapy, radiotherapy and immunotherapy, will rely on effective dosing strategies that limit the occurrence of haematological and other toxicities [53].

## Conclusions

Module 3 of this Phase 1 study demonstrated that ceralasertib plus durvalumab had a manageable safety profile with preliminary signs of antitumour activity in patients with advanced NSCLC or HNSCC. Ceralasertib 240 mg BID on Days 15–28 plus durvalumab 1500 mg on Day 1 of each 28-day cycle was the RP2D. This dosing strategy was optimised in subsequent studies in homogenous patient populations. Based on data from HUDSON and from the present study on monocyte and CD8+ T cell recovery with 7-day versus 14-day dosing, the Phase 3 LATIFY study (NCT05450692) is comparing durvalumab plus ceralasertib 240 mg BID on a 7-day dosing schedule versus docetaxel in locally advanced/metastatic NSCLC that has progressed on or after anti-PD-(L)1 treatment and platinum-based therapy [28].

### Data sharing

Data underlying the findings described in this manuscript may be obtained in accordance with AstraZeneca’s data sharing policy described at https://astrazenecagrouptrials.pharmacm.com/ST/Submission/Disclosure. Data for studies directly listed on Vivli can be requested through Vivli at www.vivli.org. Data for studies not listed on Vivli could be requested through Vivli at https://vivli.org/members/enquiries-about-studies-not-listed-on-the-vivli-platform/. AstraZeneca Vivli member page is also available outlining further details: https://vivli.org/ourmember/astrazeneca/.

## Supplementary information

Supplementary Material
