# Supplementary material for: Phase 1 study of ceralasertib, an ATR kinase inhibitor, in combination with durvalumab in patients with recurrent or metastatic NSCLC or HNSCC
Source: Br J Cancer. 2026 Mar 31;134(11):1568–79. doi: 10.1038/s41416-026-03408-y (PMC13184085; doi:10.1038/s41416-026-03408-y)
Supplement: Supplementary file 1 — Supplementary Material [file 41416_2026_3408_MOESM1_ESM.docx]

**SUPPLEMENTARY APPENDIX**

**SUPPLEMENTARY METHODS**

**Patient eligibility criteria**

Module 3 included patients aged ≥18 years with histologically or cytologically confirmed advanced recurrent or metastatic NSCLC or HNSCC and radiologically confirmed progression after at least one previous line of treatment. Primary tumour locations could include the lung, head and neck including nasopharynx, larynx, and trachea, oropharynx, hypopharynx, and oral cavity. For patients with HNSCC, the first-line regimen must have included a doublet 5-fluoropyrimidine and platinum-based regimen or immunotherapy (cetuximab was permitted). Adjuvant or neoadjuvant chemotherapy containing a doublet 5-fluoropyrimidine and platinum-based regimen was considered first-line treatment if relapse occurred within 6 months of completion. Prior radiation treatment (HNSCC) and previous concomitant or neoadjuvant chemotherapy completed more than 6 months before starting first-line therapy (NSCLC or HNSCC) were allowed. There were no further inclusion requirements regarding prior treatment for patients with NSCLC. Other key eligibility criteria were: Eastern Cooperative Oncology Group performance status 0 or 1; body weight >30 kg; estimated life expectancy >12 weeks; at least one measurable lesion ≥10 mm in the longest diameter (except lymph nodes which required a short axis ≥15 mm) that was suitable for repeated assessment; and provision of a tumour tissue sample (archival or fresh).

Patients meeting any of the following criteria were excluded: prior exposure to an ATR inhibitor; any unresolved toxicities (except alopecia) from prior therapy of National Cancer Institute Common Terminology Criteria for Adverse Events (NCI CTCAE) version 4.03 grade ≥2; spinal cord compression or brain metastases unless asymptomatic, stable and not requiring steroids for ≥4 weeks before starting study treatment; inadequate organ function, including hepatic, renal, bone marrow, and cardiac function; use of concomitant medications, herbal supplements, or foods that significantly modulate CYP3A4 or P-glycoprotein activity; active or documented autoimmune or inflammatory disease; history of organ transplant requiring use of immunosuppressive medication; receipt of live, attenuated vaccine within 30 days before first dose of durvalumab; current or prior use of immunosuppressive medication within 28 days before first dose of durvalumab (except intranasal and inhaled corticosteroids or systemic corticosteroids at physiologic doses not exceeding 10 mg/day of prednisone or equivalent).

**Biopsy cohort eligibility**

In the biopsy cohort, pre-treatment and on-treatment tumour samples were collected before the first dose of ceralasertib (during screening) and 1–6 hours post-dose on Day 5 (±1 day) of Cycle 0. Where possible, pre-treatment and on-treatment biopsies were taken from the same lesion to ensure sample consistency and reduce variability. Patients were considered evaluable for the biopsy cohort if they successfully contributed 2 mm^2^ tissue from both pre- and post-ceralasertib treatment biopsies and completed a minimum of 3 consecutive days of ceralasertib dosing in Cycle 0 immediately prior to on-treatment biopsy. A sample size of 24 evaluable patients was determined to be sufficient to assess the biological effects of ATR inhibition with ceralasertib, and 23 were enrolled to the biopsy cohort.

**Dose-escalation rules and definition of dose-limiting toxicity (DLT)**

Dose-escalation and de-escalation rules were as follows – patients were enrolled to ensure a minimum of 3 evaluable patients at the initial ceralasertib/durvalumab combination dose. Following review of safety and tolerability in the first 3 evaluable patients:

- If no dose-limiting toxicity (DLT) was observed in a cohort of 3–6 evaluable patients then dose escalation could occur; dose increases were only permitted after a review of data from a minimum of 3 evaluable patients had been conducted.
- If 1 patient experienced a DLT in a group of 3 or more evaluable patients then the cohort was to be expanded to include 6 evaluable patients.
- If 1 patient experienced a DLT in a group of 6 evaluable patients then the decision was to be made by the Safety Review Committee (SRC) to either escalate the ceralasertib dose or to expand the cohort to include 12 evaluable patients.
- If 2 or more patients experienced a DLT in a group of up to 6 evaluable patients (or 4 or more in a group of up to 12), irrespective of the number of patients enrolled, the dose was to be considered not tolerated and recruitment to the cohort and dose escalation was to cease. A lower intermediary dose (de-escalation) could potentially be considered.

After each dose level, the SRC evaluated the safety and tolerability and pharmacokinetics of ceralasertib to determine the next dose and/or schedule.

Definition of DLTs:

- DLTs were defined as the events below occurring during Cycle 0 or Cycle 1:
  - Haematological toxicities
    - Grade 4 neutropenia (absolute neutrophil count [ANC] <500 cells/mm^3^) lasting >4 consecutive days
    - Grade 3 neutropenia (ANC ≥500 to <1000 cells/mm^3^) of any duration accompanied by fever ≥38.5°C and/or systemic infection
    - Grade 3 thrombocytopenia/platelet count decreased (25,000 to <50,000/mm^3^) with bleeding
    - Any other confirmed grade ≥4 haematological toxicity.
  - Non-haematological grade ≥3 toxicities including:
    - Laboratory abnormalities
    - QTc prolongation (>500 msec)
    - Colitis (irrespective of duration).
  - Any grade 4 immune-mediated adverse event (AE), irrespective of duration.
  - Any grade 3 or 4 non-infectious pneumonitis irrespective of duration.
  - Any grade 3 immune-mediated AE, excluding colitis or pneumonitis, that does not downgrade to grade ≤2 within 3 days of onset despite maximal supportive care including systemic corticosteroids, or downgrade to grade ≤1 within 14 days of onset.
  - Any grade ≥2 pneumonitis or interstitial lung disease that does not resolve to grade 1 within 3 days of starting maximal supportive care.
  - Any other toxicity that is greater than that at baseline, and is clinically significant and/or unacceptable, and does not respond to supportive care.
  - Any event, including significant dose reductions or omissions, judged to be a DLT by the Safety Review Committee.
- DLTs excluded:
  - Alopecia of any grade.
  - Inadequately treated grade 3 nausea and/or vomiting and grade 3 diarrhoea; all patients were to receive optimal antiemetic and/or antidiarrhoeal prophylaxis and/or treatment.
  - Any toxicity clearly unrelated to the ceralasertib/durvalumab combination (e.g. solely related to the disease or disease-related process under investigation).

**SUPPLEMENTARY TABLES**

**Table S1.** Safety summary (safety analysis set)

| **AE, n (%)** | **Total**  **(N=60)** | **Cohort 1**  **(n=9)** | **Cohort 2**  **(n=3)** | **Cohort 3**  **(n=3)** | **Cohort 4**  **(n=7)** | **Cohort 5**  **(n=7)** | **Cohort 6**  **(n=8)** | **Biopsy cohort**  **(n=23)** | **Cohort 6 + Biopsy cohort* (n=31)** |
| --- | --- | --- | --- | --- | --- | --- | --- | --- | --- |
| Any AE |  |  |  |  |  |  |  |  |  |
| Treatment-emergent | 59 (98.3) | 9 (100) | 3 (100) | 3 (100) | 7 (100) | 7 (100) | 8 (100) | 22 (95.7) | 30 (96.8) |
| Treatment-related | 48 (80.0) | 8 (88.9) | 3 (100) | 3 (100) | 6 (85.7) | 5 (71.4) | 7 (87.5) | 16 (69.6) | 23 (74.2) |
| Any grade ≥3 AE |  |  |  |  |  |  |  |  |  |
| Treatment-emergent | 31 (51.7) | 3 (33.3) | 1 (33.3) | 1 (33.3) | 3 (42.9) | 3 (42.9) | 6 (75.0) | 14 (60.9) | 20 (64.5) |
| Treatment-related | 18 (30.0) | 0 | 1 (33.3) | 1 (33.3) | 1 (14.3) | 2 (28.6) | 4 (50.0) | 9 (39.1) | 13 (41.9) |
| Any SAE (incl. outcome of death) |  |  |  |  |  |  |  |  |  |
| Treatment-emergent | 23 (38.3) | 4 (44.4) | 0 | 1 (33.3) | 0 | 2 (28.6) | 5 (62.5) | 11 (47.8) | 16 (51.6) |
| Treatment-related | 7 (11.7) | 1 (11.1) | 0 | 0 | 0 | 1 (14.3) | 2 (25.0) | 3 (13.0) | 5 (16.1) |
| Any AE with outcome of death^†^ |  |  |  |  |  |  |  |  |  |
| Treatment-emergent | 4 (6.7) | 0 | 0 | 0 | 0 | 1 (14.3) | 1 (12.5) | 2 (8.7) | 3 (9.7) |
| Treatment-related | 0 | 0 | 0 | 0 | 0 | 0 | 0 | 0 | 0 |
| Any AE leading to discontinuation of ceralasertib^‡^ |  |  |  |  |  |  |  |  |  |
| Treatment-emergent | 7 (11.7) | 0 | 1 (33.3) | 0 | 0 | 1 (14.3) | 1 (12.5) | 4 (17.4) | 5 (16.1) |
| Treatment-related^§^ | 4 (6.7) | 0 | 1 (33.3) | 0 | 0 | 1 (14.3) | 1 (12.5) | 1 (4.3) | 2 (6.5) |

Patients received ceralasertib at doses of 80 mg BID (Cohort 1), 160 mg BID (Cohort 2), 320 mg QD (Cohorts 3 and 4), and 240 mg BID (Cohorts 5 and 6, and the biopsy cohort) in combination with a fixed dose of durvalumab (1500 mg IV Q4W); the number of dosing days per cycle varied between cohorts receiving the same daily dose of ceralasertib (see **Supplementary Figure S1**).

Relatedness to study treatment was as assessed by the investigator.

*RP2D: data pooled from Cohort 6 and biopsy cohort.

^†^AEs leading to death were cardiorespiratory arrest (n=1; biopsy cohort), haemoptysis (n=1; Cohort 5), pneumonia (n=1; Cohort 6); and pulmonary haemorrhage (n=1; biopsy cohort).

^‡^AEs leading to discontinuation of ceralasertib that were considered related to durvalumab only were Guillain-Barré syndrome (n=1; cohort 5); immune-mediated neuropathy (n=1; cohort 6); and pneumonitis (n=1; biopsy cohort). The AEs also led to discontinuation of durvalumab in these patients.

^§^One AE (musculoskeletal pain) considered related to both ceralasertib and durvalumab led to discontinuation of both drugs in a patient in Cohort 2.

AE, adverse event; BID, twice daily; IV, intravenous; Q4W, every 4 weeks; QD, once daily; SAE, serious adverse event.

**Table S2.** Treatment-emergent SAEs (safety analysis set)

| **SAE, n (%)** | **Total**  **(N=60)** | **Cohort 1**  **(n=9)** | **Cohort 2**  **(n=3)** | **Cohort 3**  **(n=3)** | **Cohort 4**  **(n=7)** | **Cohort 5**  **(n=7)** | **Cohort 6**  **(n=8)** | **Biopsy cohort**  **(n=23)** | **Cohort 6 + Biopsy cohort* (n=31)** |
| --- | --- | --- | --- | --- | --- | --- | --- | --- | --- |
| Any | 23 (38.3) | 4 (44.4) | 0 | 1 (33.3) | 0 | 2 (28.6) | 5 (62.5) | 11 (47.8) | 16 (51.6) |
| Lower respiratory tract infection | 5 (8.3) | 2 (22.2) | 0 | 0 | 0 | 0 | 2 (25.0) | 1 (4.3) | 3 (9.7) |
| Thrombocytopenia/platelet count decreased | 3 (5.0) | 0 | 0 | 0 | 0 | 0 | 1 (12.5) | 2 (8.7) | 3 (9.7) |
| Atrial fibrillation | 2 (3.3) | 0 | 0 | 0 | 0 | 0 | 0 | 2 (8.7) | 2 (6.5) |
| Dyspnoea | 2 (3.3) | 1 (11.1) | 0 | 0 | 0 | 1 (14.3) | 0 | 0 | 0 |
| Haemoptysis | 2 (3.3) | 0 | 0 | 0 | 0 | 1 (14.3) | 0 | 1 (4.3) | 1 (3.2) |
| Pneumonia | 2 (3.3) | 0 | 0 | 0 | 0 | 0 | 2 (25.0) | 0 | 2 (6.5) |
| Urinary tract infection | 2 (3.3) | 0 | 0 | 0 | 0 | 0 | 0 | 2 (8.7) | 2 (6.5) |
| Abdominal pain | 1 (1.7) | 0 | 0 | 0 | 0 | 0 | 0 | 1 (4.3) | 1 (3.2) |
| Aphasia | 1 (1.7) | 0 | 0 | 0 | 0 | 0 | 1 (12.5) | 0 | 1 (3.2) |
| Ascites | 1 (1.7) | 0 | 0 | 0 | 0 | 0 | 0 | 1 (4.3) | 1 (3.2) |
| Autoimmune arthritis | 1 (1.7) | 0 | 0 | 0 | 0 | 0 | 1 (12.5) | 0 | 1 (3.2) |
| Cardiorespiratory arrest | 1 (1.7) | 0 | 0 | 0 | 0 | 0 | 0 | 1 (4.3) | 1 (3.2) |
| Chronic inflammatory demyelinating polyradiculoneuropathy | 1 (1.7) | 0 | 0 | 0 | 0 | 1 (14.3) | 0 | 0 | 0 |
| COPD | 1 (1.7) | 0 | 0 | 0 | 0 | 0 | 0 | 1 (4.3) | 1 (3.2) |
| Constipation | 1 (1.7) | 0 | 0 | 0 | 0 | 1 (14.3) | 0 | 0 | 0 |
| Diarrhoea | 1 (1.7) | 0 | 0 | 0 | 0 | 0 | 0 | 1 (4.3) | 1 (3.2) |
| Guillain-Barré syndrome | 1 (1.7) | 0 | 0 | 0 | 0 | 1 (14.3) | 0 | 0 | 0 |
| Hypokalaemia | 1 (1.7) | 1 (11.1) | 0 | 0 | 0 | 0 | 0 | 0 | 0 |
| Immune-mediated neuropathy | 1 (1.7) | 0 | 0 | 0 | 0 | 0 | 1 (12.5) | 0 | 1 (3.2) |
| Malnutrition | 1 (1.7) | 0 | 0 | 0 | 0 | 0 | 0 | 1 (4.3) | 1 (3.2) |
| Myocardial infarction | 1 (1.7) | 0 | 0 | 0 | 0 | 0 | 0 | 1 (4.3) | 1 (3.2) |
| Neutropenia | 1 (1.7) | 0 | 0 | 0 | 0 | 0 | 1 (12.5) | 0 | 1 (3.2) |
| Non-cardiac chest pain | 1 (1.7) | 0 | 0 | 0 | 0 | 0 | 0 | 1 (4.3) | 1 (3.2) |
| Pneumocystis jirovecii pneumonia | 1 (1.7) | 0 | 0 | 0 | 0 | 0 | 1 (12.5) | 0 | 1 (3.2) |
| Pneumonia aspiration | 1 (1.7) | 1 (11.1) | 0 | 0 | 0 | 0 | 0 | 0 | 0 |
| Pneumonitis | 1 (1.7) | 0 | 0 | 0 | 0 | 0 | 0 | 1 (4.3) | 1 (3.2) |
| Pneumothorax | 1 (1.7) | 0 | 0 | 1 (33.3) | 0 | 0 | 0 | 0 | 0 |
| Pulmonary embolism | 1 (1.7) | 0 | 0 | 0 | 0 | 0 | 0 | 1 (4.3) | 1 (3.2) |
| Pulmonary haemorrhage | 1 (1.7) | 0 | 0 | 0 | 0 | 0 | 0 | 1 (4.3) | 1 (3.2) |
| Pyrexia | 1 (1.7) | 0 | 0 | 0 | 0 | 0 | 0 | 1 (4.3) | 1 (3.2) |
| Urinary retention | 1 (1.7) | 0 | 0 | 0 | 0 | 0 | 0 | 1 (4.3) | 1 (3.2) |
| Viral upper respiratory tract infection | 1 (1.7) | 1 (11.1) | 0 | 0 | 0 | 0 | 0 | 0 | 0 |

Patients received ceralasertib at doses of 80 mg BID (Cohort 1), 160 mg BID (Cohort 2), 320 mg QD (Cohorts 3 and 4), and 240 mg BID (Cohorts 5 and 6, and the biopsy cohort) in combination with a fixed dose of durvalumab (1500 mg intravenously every 4 weeks); the number of dosing days per cycle varied between cohorts receiving the same daily dose of ceralasertib (see **Supplementary Figure S1**). Includes SAEs with an onset date on or after the date of first dose of study drug (ceralasertib or durvalumab), up to and including 90 days following the date of last dose of study drug. *RP2D: data pooled from Cohort 6 and biopsy cohort. BID, twice daily; COPD, chronic obstructive pulmonary disease; QD, once daily, SAE, serious adverse event.

**Table S3.** Summary of ceralasertib plasma pharmacokinetic parameters after a single dose of ceralasertib monotherapy (Cycle 0, Day 1) and at steady state (Cycle 0, Day 5 or 8).

|  | | **Ceralasertib monotherapy dose [Cohort(s)]** | | | | | | | |
| --- | --- | --- | --- | --- | --- | --- | --- | --- | --- |
|  |  | **80 mg BID [Cohort 1]**  **(n=9)** | | **160 mg BID [Cohort 2]**  **(n=3)** | | **240 mg BID [Cohorts 5, 6, Biopsy cohort]**  **(n=36/n=29)*** | | **320 mg QD [Cohorts 3 & 4]**  **(n=10)** | |
| **Parameter** | **Statistic** | **Single dose** | **Steady state** | **Single dose** | **Steady state** | **Single dose** | **Steady state** | **Single dose** | **Steady state** |
| C_max_, ng/mL | n | 7 | 8 | 3 | 2 | 35 | 25 | 8 | 9 |
|  | Geomean (CV%) | 3144 (53.63) | 5357 (35.11) | 6016 (13.69) | NC | 8178 (33.91) | 10,940 (31.09) | 8336 (26.46) | 9184 (22.52) |
| t_max_, h | n | 7 | 8 | 3 | 2 | 35 | 25 | 8 | 9 |
|  | Median  [Min–Max] | 1.03  [0.50–2.03] | 1.01  [0.50–2.00] | 1.15  [1.00–2.00] | NC  [1.00–1.00] | 1.05  [0.48–4.00] | 1.03  [0.42–6.00] | 1.51  [0.50–4.03] | 1.10  [0.92–4.00] |
| AUC_(0-6)_, h*ng/mL | n | 7 | 8 | 3 | 2 | 33 | 24 | 7 | 8 |
|  | Geomean (CV%) | 13,210 (46.36) | 24,140 (44.87) | 25,070 (15.81) | NC | 35,300 (35.59) | 51,960 (38.03) | 38,630 (19.78) | 38,270 (25.43) |
| AUC_(0-8)_, h*ng/mL | n | 6 | 8 | 3 | 2 | 25 | 13 | 7 | 8 |
|  | Geomean (CV%) | 19,110 (22.13) | 29,910 (48.20) | 30,190 (16.33) | NC | 44,020 (36.39) | 58,250 (38.44) | 48,760 (20.57) | 47,860 (28.33) |
| C_max_/D, ng/mL/mg | n | 7 | 8 | 3 | 2 | 35 | 25 | 8 | 9 |
|  | Geomean (CV%) | 39.30 (53.63) | 66.96 (35.11) | 37.60 (13.69) | NC | 34.08 (33.91) | 45.58 (31.09) | 26.05 (26.46) | 28.70 (22.52) |
| AUC_(0-6)_/D, h*ng/mL/mg | n | 7 | 8 | 3 | 2 | 33 | 24 | 7 | 8 |
|  | Geomean (CV%) | 165.1 (46.36) | 301.7 (44.87) | 156.7 (15.81) | NC | 147.1 (35.59) | 216.5 (38.03) | 120.7 (19.78) | 119.6 (25.43) |
| AUC_(0-8)_/D, h*ng/mL/mg | n | 6 | 8 | 3 | 2 | 25 | 13 | 7 | 8 |
|  | Geomean (CV%) | 238.9 (22.13) | 373.8 (48.20) | 188.7 (16.33) | NC | 183.4 (36.39) | 242.7 (38.44) | 152.4 (20.57) | 149.6 (28.33) |
| R_ac_(C_max_) | n | NA | 7 | NA | 2 | NA | 24 | NA | 7 |
|  | Geomean (CV%)  [Min–Max] | NA | 1.896 (55.35)  [1.24–5.58] | NA | NC  [1.39–1.50] | NA | 1.327 (18.03)  [0.962–1.99] | NA | 1.133 (15.95)  [0.849–1.27] |
| R_ac_(AUC_(0-6)_) | n | NA | 7 | NA | 2 | NA | 22 | NA | 6 |
|  | Geomean (CV%)  [Min–Max] | NA | 2.070 (48.61)  [1.44–5.67] | NA | NC  [1.54–1.58] | NA | 1.439 (16.85)  [1.08–2.19] | NA | 1.151 (17.48)  [0.877–1.33] |

*The single dose parameters are for patients from Cohorts 5 and 6 and the biopsy cohort (n=36); the steady-state parameters are for patients from Cohort 6 and the biopsy cohort only (n=29).

AUC, area under the plasma concentration–time curve; BID, twice daily; C_max_, maximum plasma concentration; CV, geometric coefficient of variance; D, dose; Geomean: geometric mean; Max, maximum; Min, minimum; NA, not applicable; NC, not calculable; QD, once daily; R_ac_(C_max_), accumulation ratio based on C_max_; R_ac_(AUC_(0-6)_), accumulation ratio based on AUC_(0-6)_; t_max_, time to maximum plasma concentration.

**Table S4**. Summary of ceralasertib plasma pharmacokinetic parameters at steady state (Cycle 1, Day XX) for ceralasertib in combination with durvalumab.

|  | | **Ceralasertib dose in combination with** **durvalumab 1500 mg Q4W [Cohort(s)]** | | | |
| --- | --- | --- | --- | --- | --- |
| **Parameter** | **Statistic** | **80 mg BID [Cohort 1]**  **(n=9)** | **160 mg BID [Cohort 2]**  **(n=3)** | **240 mg BID [Cohorts 5 & 6]**  **(n=15)** | **320 mg QD [Cohorts 3 & 4]**  **(n=10)** |
| C_max_, ng/mL | n | 7 | 2 | 12 | 9 |
|  | Geomean (CV%)  [Min–Max] | 5254 (56.88)  [2590–13,100] | NC  [8320–9690] | 10,780 (27.54)  [6530–16,300] | 9115 (12.80)  [7910–11,900] |
| t_max_, h | n | 7 | 2 | 12 | 9 |
|  | Median [Min–Max] | 1.00 [0.47–2.00] | NC [0.50–1.00] | 1.38 [0.5–2.00] | 1.00 [0.48–2.00] |
| AUC_(0–6)_, h*ng/mL | n | 7 | 2 | 12 | 9 |
|  | Geomean (CV%)  [Min–Max] | 22,990 (69.63)  [10,300–72,000] | NC  [42,000–43,300] | 52,130 (33.05)  [28,800–75,200] | 39,210 (23.41)  [28,600–53,500] |
| AUC_(0–8)_, h*ng/mL | n | 7 | 2 | 12 | 8 |
|  | Geomean (CV%)  [Min–Max] | 28,560 (74.07)  [12,400–95,800] | NC  [53,600–54,300] | 64,980 (35.33)  [34,200–98,000] | 47,330 (24.65)  [34,500–69,900] |
| C_max_/D, ng/mL/mg | n | 7 | 2 | 12 | 9 |
|  | Geomean (CV%)  [Min–Max] | 65.68 (56.88)  [32.4–164] | NC  [52.0–60.6] | 44.91 (27.54)  [27.2–67.9] | 28.48 (12.80)  [24.7–37.2] |
| AUC_(0–6)_/D, h*ng/mL/mg | n | 7 | 2 | 12 | 9 |
|  | Geomean (CV%)  [Min–Max] | 287.4 (69.63)  [129–900] | NC  [263–271] | 217.2 (33.05)  [120–313] | 122.5 (23.41)  [89.4–167] |
| AUC_(0–8)_/D, h*ng/mL/mg | n | 7 | 2 | 12 | 8 |
|  | Geomean (CV%)  [Min–Max] | 357.0 (74.07)  [155–1200] | NC  [335–340] | 270.8 (35.33)  [142–408] | 147.9 (24.65)  [108–218] |

AUC, area under the plasma concentration–time curve; BID, twice daily; C_max_, maximum plasma concentration; CV, geometric coefficient of variance; D, dose; Geomean: geometric mean; Max, maximum; Min, minimum; NC, not calculable; Q4W, every 4 weeks; QD, once daily; t_max_, time to maximum plasma concentration.

**SUPPLEMENTARY FIGURES**

**Figure S1. Dosing schedule for ceralasertib in combination with durvalumab.**


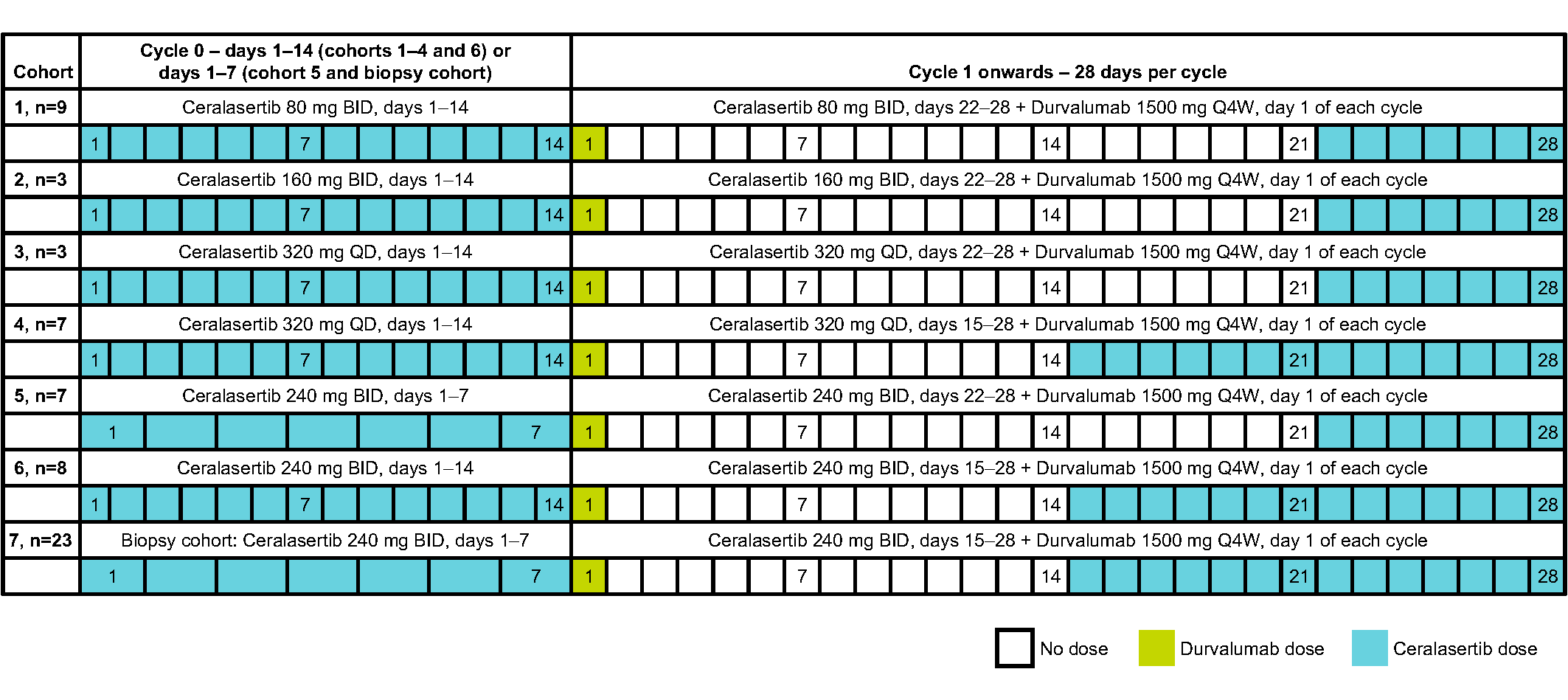


BID, twice daily; Q4W, every four weeks; QD, once daily.

**Figure S2. Pharmacokinetic and pharmacodynamic blood sample collection schedule for cohorts 1–6 and the biopsy cohort.**


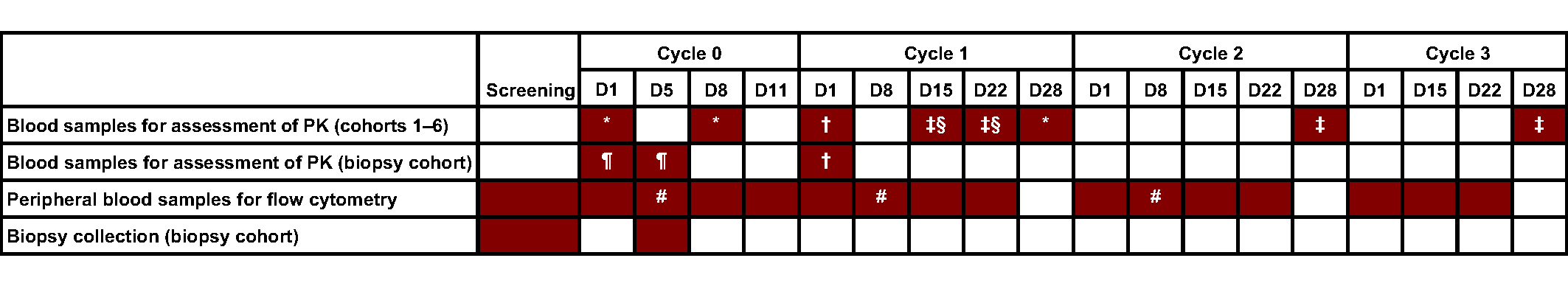


*Samples collected prior to, and 0.5, 1, 2, 4, and 8 h after ceralasertib dose.

^†^Sample collected before durvalumab dose. ^‡^Sample collected before ceralasertib dose.

^§^Sample collected on first day of ceralasertib dosing in Cycle 1 (Day 15 or Day 22, cohort-dependent)

^¶^Samples collected prior to, and 0.5, 1, 2, 4, and 6 h after ceralasertib dose.

^#^Biopsy cohort only.

PK, pharmacokinetics.

**Figure S3. Effects of ceralasertib on platelet and neutrophil counts.** Effect of ceralasertib 240 mg BID, Days 15–28, on platelet counts (**a**) and on neutrophil counts (**b**), and of ceralasertib 240 mg BID, Days 22–28, on platelet counts (**c**) and on neutrophil counts (**d**).


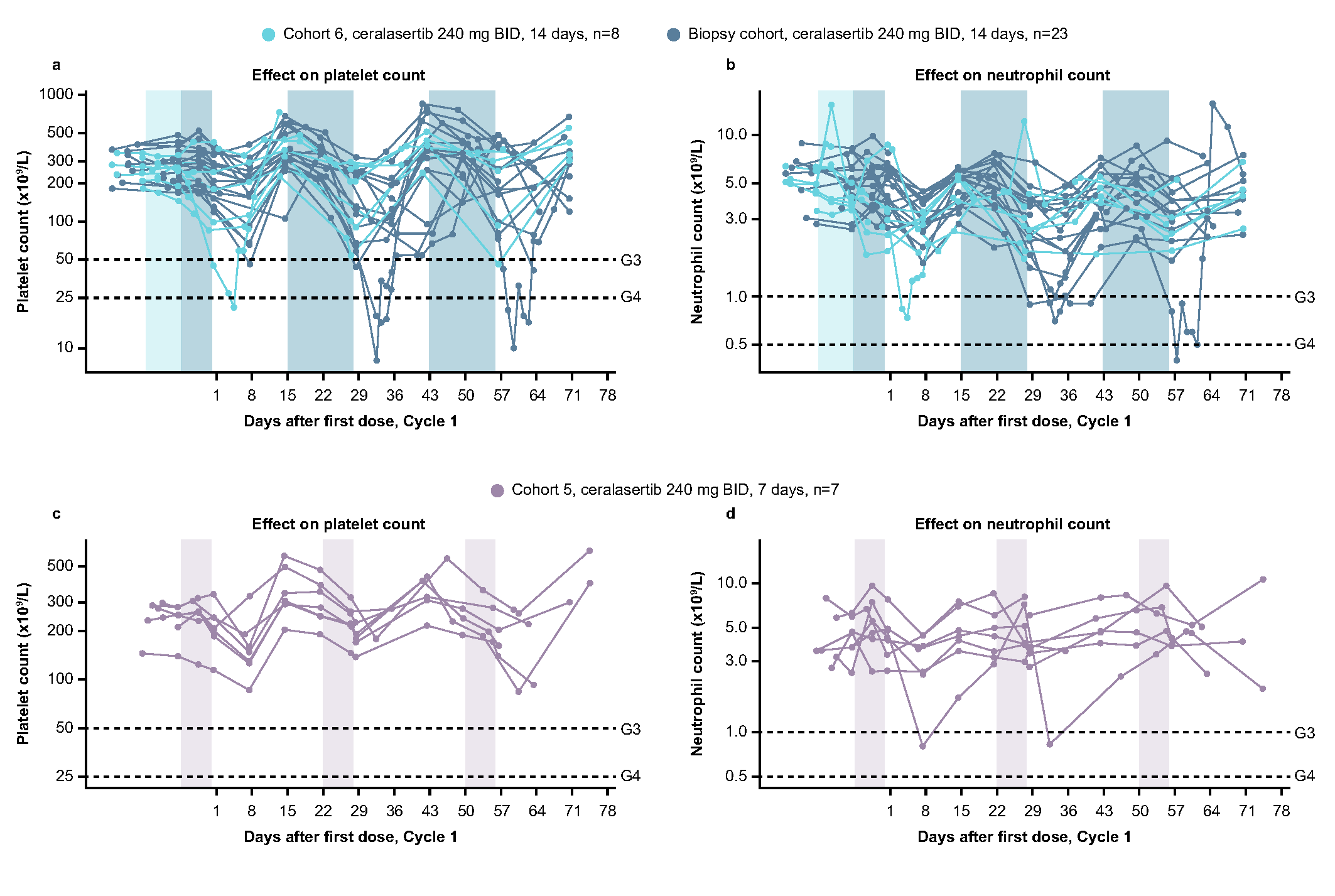


Shaded rectangles represent ceralasertib administration times. Cohort 6 received a 14-day Cycle 0 of ceralasertib treatment and the biopsy cohort received a 7-day Cycle 0 of ceralasertib treatment.

BID, twice daily; G3, grade 3; G4, grade 4.

**Figure S4. Time to and duration of objective tumour responses.**


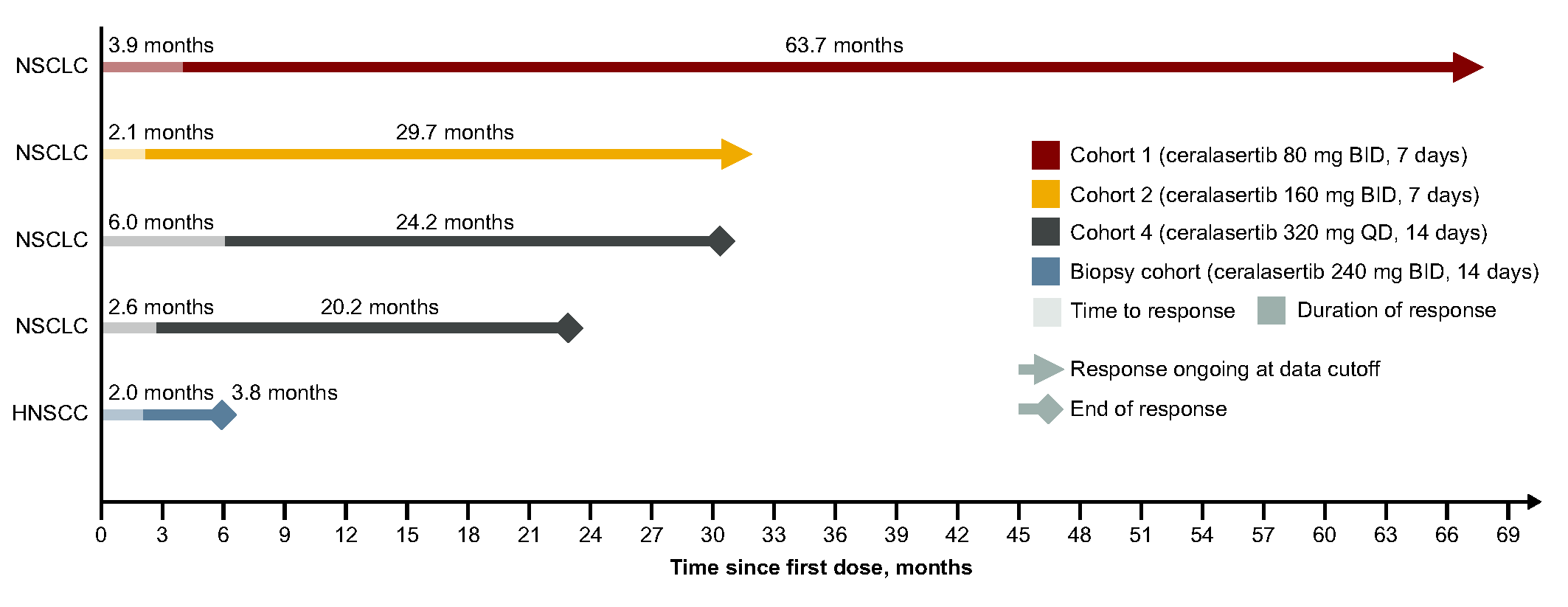


BID, twice daily; HNSCC, head and neck squamous cell carcinoma; NSCLC, non-small-cell lung cancer; QD, once daily.

**Figure S5. pRAD50 expression in pre- and post-treatment tumour samples in the biopsy cohort (ATM >0%).** IHC staining (**a**) and quantification of individual patients’ paired samples (**b**).


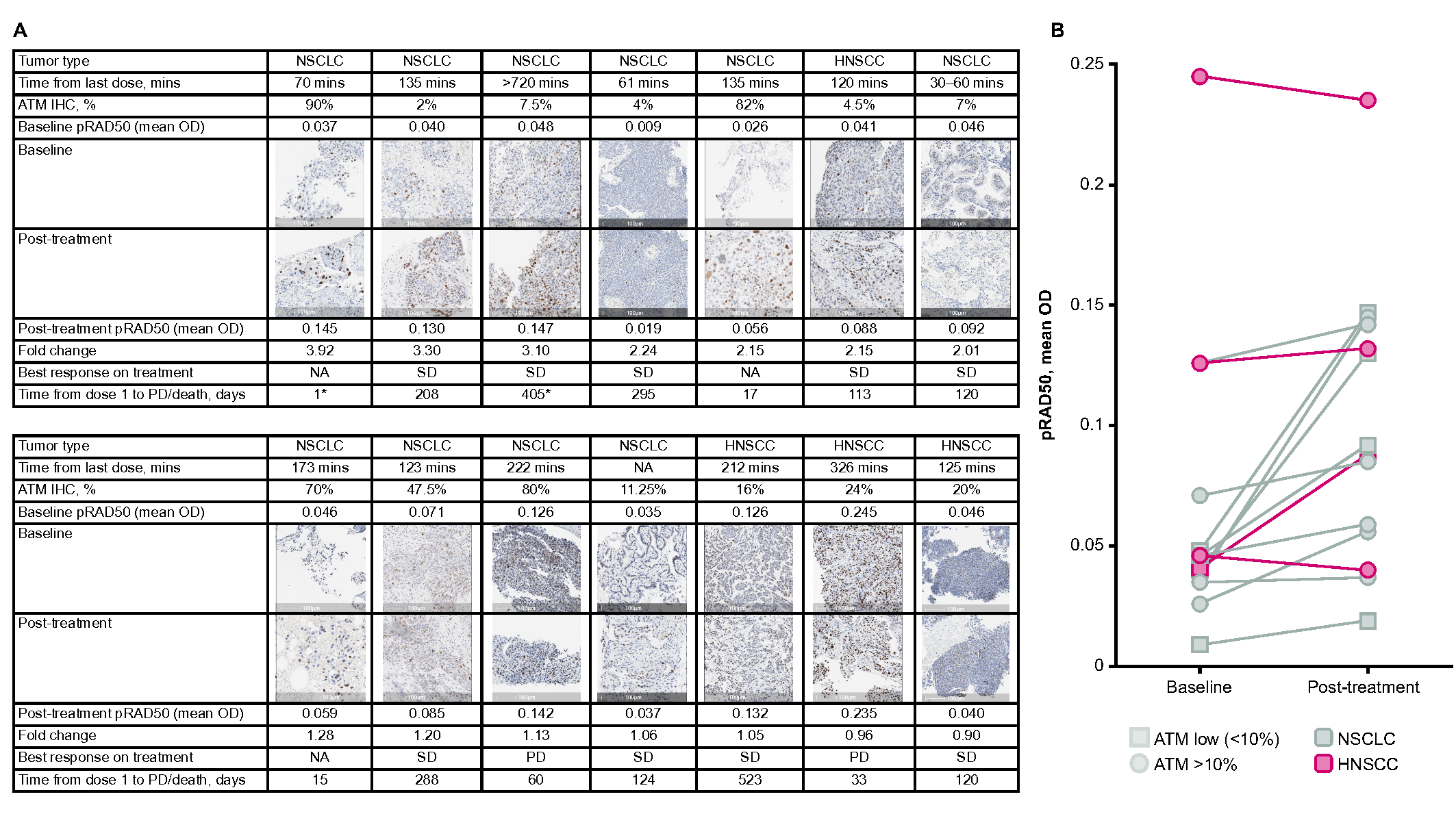


Biopsies taken predose at screening and postdose on Day 5 (+/– 1 day) of ceralasertib 240 mg BID monotherapy. ATM IHC % expression is an average of multiple samples (up to 4 cores across the two time-points) for each individual patient, with at least one biopsy having ≥10% ATM expression. *Censored, no progression event.

ATM, ataxia telangiectasia mutated; HNSCC, head and neck squamous cell carcinoma; IHC, immunohistochemistry; NSCLC, non-small-cell lung cancer; OD, optical density; PD, progressive disease.

**Figure S6. Monocyte and proliferating T-cell pharmacodynamics.** Monocyte modulation (**a**) and proliferating CD8+ Ki67+ T-cell modulation (**b**) with 14 days of ceralasertib dosing in Cycle 0, and monocyte modulation (**c**) and proliferating CD8+ T-cell modulation (**d**) with ceralasertib 240 mg BID for 7 or 14 days in Cycle 0.


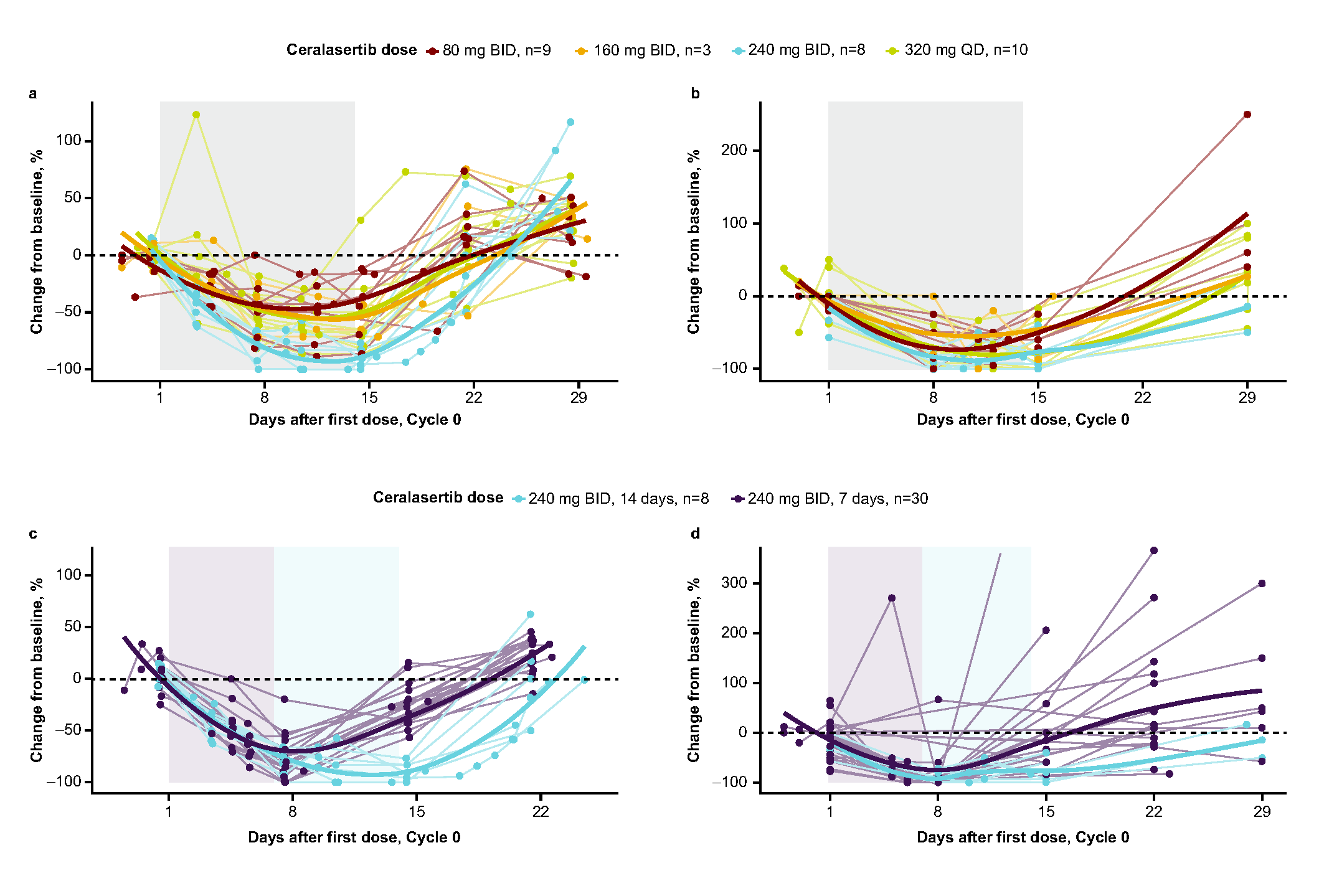


Baseline T-cell values consisted of the mean between samples at screening and Cycle 0 Day 1 (biomarker values equal to 0 during the screening period were excluded from the change from baseline calculation). Shaded areas represent periods of ceralasertib dosing.

BID, twice daily; QD, once daily.

**Figure S7. CD8+ T-cell pharmacodynamics observed across each cohort.** T-cell rebound was seen in all cohorts not only in Cycle 0 but also in subsequent cycles.


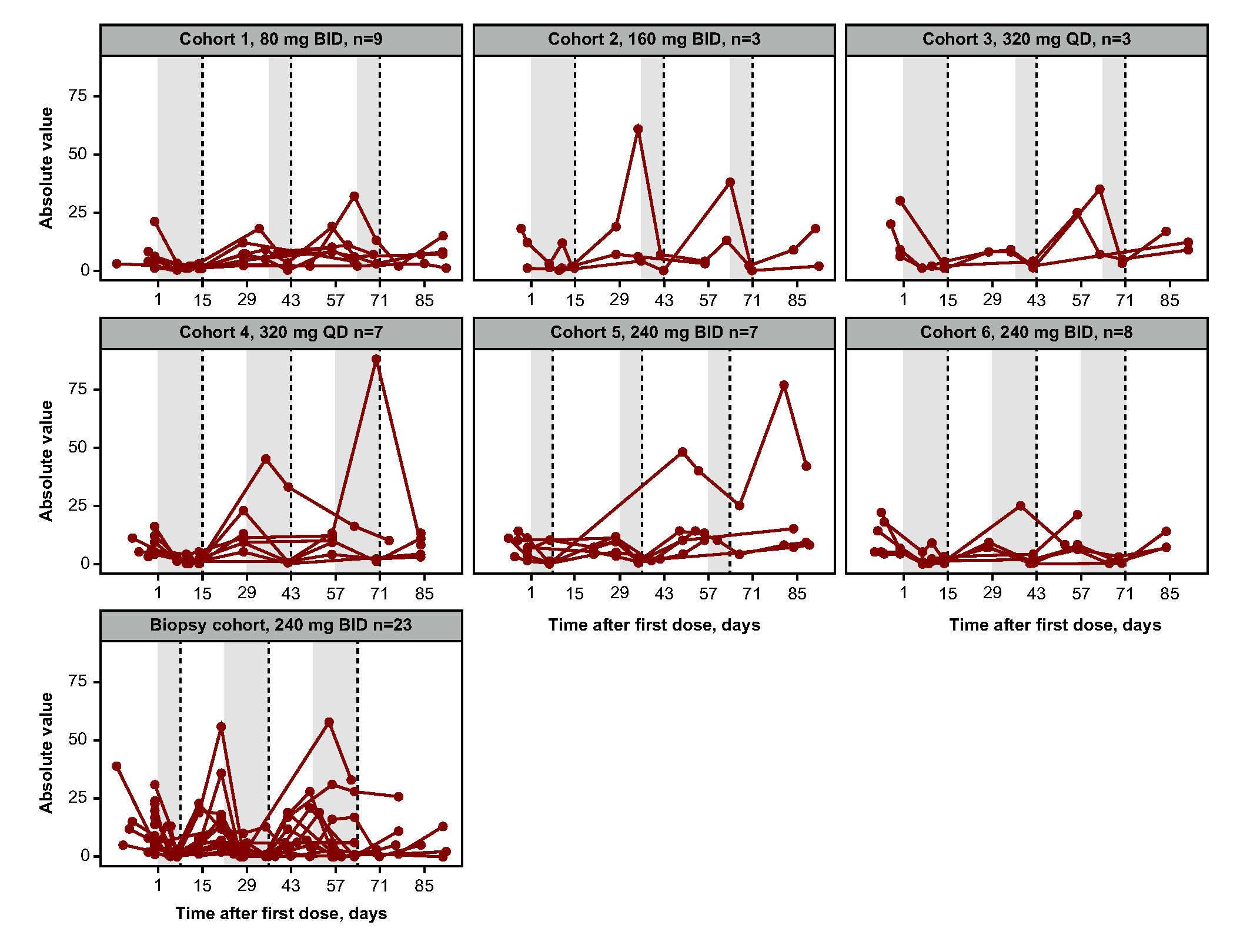


Dashed lines represent the ends of Cycles 0, 1 and 2, respectively. Cohorts 1–4 and Cohort 6 received a 14-day Cycle 0 (ceralasertib monotherapy); Cohort 5 and the biopsy cohort received a 7-day Cycle 0 (ceralasertib monotherapy). Shaded areas represent periods of ceralasertib dosing.

Patients received ceralasertib at doses of 80 mg BID (Cohort 1), 160 mg BID (Cohort 2), 320 mg QD (Cohorts 3 and 4), and 240 mg BID (Cohorts 5 and 6, and the biopsy cohort) in combination with a fixed dose of durvalumab (1500 mg intravenously every 4 weeks); the number of dosing days per cycle varied between cohorts receiving the same daily dose of ceralasertib (see **Supplementary Figure S1**).

BID, twice daily; IV, intravenous; Q4W, every 4 weeks; QD, once daily.
